# Sulfisoxazole inhibits the secretion of small extracellular vesicles by targeting the endothelin receptor A

**DOI:** 10.1038/s41467-019-09387-4

**Published:** 2019-03-27

**Authors:** Eun-Ju Im, Chan-Hyeong Lee, Pyong-Gon Moon, Gunassekaran Gowri Rangaswamy, Byungheon Lee, Jae Man Lee, Jae-Chul Lee, Jun-Goo Jee, Jong-Sup Bae, Taeg-Kyu Kwon, Keon-Wook Kang, Myeong-Seon Jeong, Joo-Eun Lee, Hyun-Suk Jung, Hyun-Joo Ro, Sangmi Jun, Wonku Kang, Seung-Yong Seo, Young-Eun Cho, Byoung-Joon Song, Moon-Chang Baek

**Affiliations:** 1 Department of Molecular Medicine, CMRI, Exosome Convergence Research Center (ECRC), School of Medicine, Kyungpook National University, Daegu, 41944 Republic of Korea; 2 Department of Biochemistry and Cell Biology, School of Medicine, Kyungpook National University, Daegu, 41944 Republic of Korea; 3 Department of Microbiology, School of Medicine, Kyungpook National University, Daegu, 41944 Republic of Korea; 4 Research Institute of Pharmaceutical Researches, College of Pharmacy, Kyungpook National University, Daegu, 41566 Republic of Korea; 5 Department of Immunology and School of Medicine, Keimyung University, Daegu, 42601 Republic of Korea; 6 College of Pharmacy and Research Institute of Pharmaceutical Sciences, Seoul National University, Seoul, 08826 Republic of Korea; 7 Chuncheon Center, Korean Basic Science Institute, Gangwon-do, 24341 Republic of Korea; 8 Department of Biochemistry, College of Natural Sciences, Kangwon National University, Gangwon-do, 24341 Republic of Korea; 9 Drug & Disease Target Group, Korea Basic Science Institute, Cheongju, 28119 Republic of Korea; 10 College of Pharmacy, Chung-Ang University, Seoul, 06974 Republic of Korea; 11 College of Pharmacy and Gachon Institute of Pharmaceutical Sciences, Gachon University, Incheon, 21936 Republic of Korea; 12 Section of Molecular Pharmacology and Toxicology, Laboratory of Membrane Biochemistry and Biophysics, National Institute on Alcohol Abuse and Alcoholism (NIAAA), Bethesda, 20892 MD USA

## Abstract

Inhibitors of the secretion of cancer exosomes, which promote cancer progression and metastasis, may not only accelerate exosome biology research but also offer therapeutic benefits for cancer patients. Here we identify sulfisoxazole (SFX) as an inhibitor of small extracellular vesicles (sEV) secretion from breast cancer cells through interference with endothelin receptor A (ETA). SFX, an FDA-approved oral antibiotic, showed significant anti-tumor and anti-metastatic effects in mouse models of breast cancer xenografts, the reduced expression of proteins involved in biogenesis and secretion of sEV, and triggered co-localization of multivesicular endosomes with lysosomes for degradation. We demonstrate the important role of ETA, as target of SFX, by gain- and loss-of-function studies of the ETA protein, through a direct binding assay, and pharmacological and genetic approaches. These findings may provide a foundation for sEV-targeted cancer therapies and the mechanistic studies on sEV biology.

## Abstract (web summary)

Extracellular vesicles are released from cells and permit communication between different cell types. Here, the authors identify that the FDA approved antibiotic sulfisoxazole, can block the release of these vesicles in breast cancer cells resulting in reduced cell growth in vitro and in vivo.

## Introduction

Metastasis is the main cause of mortality in cancer patients, but clinical options against advanced metastasis stage of cancer remain limited owing to high complexity of the biological events of metastasis, leading to inefficient drug development and poor treatment outcomes^1,2^. Exosomes are 50–150 nm small extracellular vesicles (sEV) that harbor proteins, lipids, RNAs, and DNA, and thereby act as important mediators of cell–cell communications in various physiological and pathological pathways^3^. Cancer-cell-derived sEV prepare a favorable microenvironment at future metastatic sites as well as the primary tumor^4–7^. Hence, the clearance of these malicious sEV in circulating system has emerged as a novel and potentially useful therapeutic strategy for anti-metastatic drug development^8^. Many reports have already demonstrated that the reduction of sEV secretion (or secreted sEV), achieved by using a chemical inhibitor^9,10^, genetic engineering^11^, or antibody^12^, can enhance the efficiency of cancer chemotherapy and inhibit cancer metastasis. However, further work is required to determine whether these inhibitors can affect the secretion of other EVs or soluble proteins, or the pathophysiological features of donor cells, as reviewed previously^13^. Moreover, the underlying mechanisms of the already-identified inhibitors that have been demonstrated to control exosome biogenesis and secretion have still not been clearly elucidated while their safety/toxicity profiles are unknown.

Drug repurposing, the process of finding new indications for existing drugs, is a faster, cheaper, and safer drug development strategy. In this process, the new indication can be derived from the same target (on-target) or a newly-recognized target (off-target) of the original drug^14^. A significant advantage of drug repurposing is that regulatory agency-approved drugs have already passed toxicity and safety tests in humans. One of major concerns for the development of a new drug to inhibit the secretion of sEV is the toxicity, probably caused by any partial or temporary inhibition of exosome secretion from normal cells when a drug candidate inhibits the secretion of sEV from cancer cells. We believe that drug repurposing could reduce the risk of failure by saving valuable time and efforts during the identification and development of a new inhibitor of sEV secretion as a novel anti-cancer therapeutic agent.

In this study, by screening the library of FDA-approved drugs, we identified sulfisoxazole (SFX), an oral antibacterial drug, as a specific inhibitor of the biogenesis and secretion of sEV from breast cancer cells, resulting in the effective suppression of breast cancer growth and metastasis without significant toxicity. Furthermore, we found that endothelin receptor A (ETA), a member of GPCR family, is critically associated with sEV biogenesis and secretion in breast cancer cells, and that ETA is a newly-identified target (off-target) of SFX, as evidenced by gain- and loss-of-function studies of the ETA protein through pharmacological and genetic approaches. Our findings may provide a foundation for sEV-targeted cancer therapies and the mechanistic studies on sEV biology.

## Results

### Discovery of a drug for inhibition of EV secretion

**Fig. 1:**
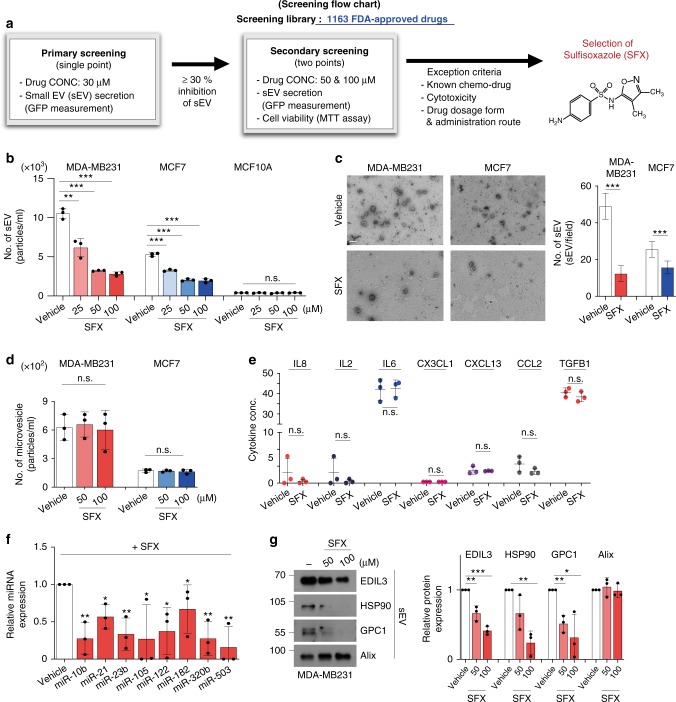
SFX inhibits the secretion of sEV from breast cancer cells quantitatively and qualitatively. **a** Screening flow chart of primary and secondary screenings with some exclusion criteria to identify an inhibitor of sEV secretion, sulfisoxazole. **b** The number of secreted sEV with the indicated concentrations of SFX. *n* *=* 3. **c** Left, electron microscopy image. Scale bar, 100 nm. Right, Quantification of the number of secreted sEV. Randomized fields were captured and counted. *n* *=* 30. **d** Measurement of the microvesicle concentration by nanoparticle tracking analysis (NTA). *n* *=* 3. **e** Measurement of soluble cytokines secreted from MDA-MB231 cells. *n* *=* 3. **f** qRT-PCR analysis of the indicated miRNAs in MDA-MB231 cells after treatment of 100 μM SFX. *n* *=* 3. The GEO accession number of miRNA microarray set is GSE124320. **g** Immunoblot of various proteins in MDA-MB231 cell-derived sEV. Equal amounts of sEV protein (10 μg) were loaded per lane. *n* *=* 3. Experiments were performed with 95% confluent cells. Significance was determined using an unpaired two-tailed Student’s *t-* test. ***p < 0.001, **p < 0.005, and *p < 0.05. Error bar, SD. Source data (**b**–**g**) are provided as a Source Data file

To identify drugs that reduce sEV secretion, we developed cell-based high-throughput assay system with 1163 FDA-approved drugs, according to the flow chart for primary and secondary screenings (Fig. 1a). To accomplish this task, MDA-MB231 triple-negative human breast cancer cells were engineered to stably secrete sEV that contain CD63-GFP (MDA-MB231 CD63-GFP (+)) and grown in 96-well plates (Supplementary Fig. 1a–h). Inhibitory effect on sEV secretion was determined by decreased fluorescence from the individual culture supernatant, which should contain sEV secreted from the cancer cells treated with each drug. During the initial primary screening, we tested drugs at 30 μM, and the top 26 drugs (that inhibited sEV secretion by up to 30%) were selected as potential candidates for the secondary assessment at 50 and 100 μM concentrations (Supplementary Fig. 2a, b). However, some drugs were not further studied based on a range of exclusion criteria, as described (Fig. 1a). Finally, the antibiotic sulfisoxazole (SFX) was selected for further in-depth evaluation because it is a cheap, orally administered FDA-approved drug without cytotoxicity at effective doses (Fig. 1a and Supplementary Fig. 2b). This drug was originally known to be a competitive inhibitor of the enzyme dihydropteroate synthetase through preventing the condensation of pteridine with *p*-aminobenzoic acid, a substrate of the enzyme in prokaryotic systems^15^.

### SFX inhibits sEV secretion quantitatively and qualitatively

We systematically analyzed the inhibitory effects of SFX on the secretion of sEV in three representative human breast cell lines: MCF10A (normal), MCF7 (weakly invasive), and MDA-MB231 (highly invasive). Notably, SFX treatment quantitatively inhibited sEV secretion from MCF7 and MDA-MB231 cancer cells, as determined by the ultracentrifugation method (Supplementary Fig. 3a, b), in a dose-dependent manner without morphological change or cytotoxic effect (Fig. 1b, c and Supplementary Fig. 3c–e). However, no significant inhibition was observed in normal MCF10A cells, which rarely secrete sEV, suggesting that SFX shows a strong inhibitory effect on the secretion of sEV from breast cancer cells (Fig. 1b and Supplementary Fig. 3c). Consistent with this reduction of secreted sEV, the amounts of sEV proteins, such as CD9, filotillin-1, Alix, Tsg101, and CD63, were decreased (Supplementary Fig. 3f). Moreover, the number of sEV and amounts of sEV proteins were reduced in SK-MEL-28 human melanoma cancer cells, which secrete a large number of sEV (Supplementary Fig. 3g).

Next, we investigated whether SFX affects other secretion pathways, such as microvesicles (MVs) and various soluble protein secretions. SFX neither significantly affected the secretion of MVs from cells nor altered the activity of acidic sphingomyelinase (aSMase), which is involved in the formation of large MV^16^ (Fig. 1d and Supplementary Fig. 3h). In addition, the classical secretion pathway was not significantly affected by SFX treatment (Fig. 1e). Furthermore, we performed miRNA microarray (Supplementary Fig. 4a) and proteomics analyses (Supplementary Fig. 4b) of MDA-MB231 sEV, and confirmed that SFX affected the components of sEV cargo, including various miRNAs and proteins (EDIL3, HSP90, and GPC1^17–19^), known to be present in sEV derived from MDA-MB231 cells^20^ (Fig. 1f, g).

### SFX inhibits breast cancer progression

**Fig. 2:**
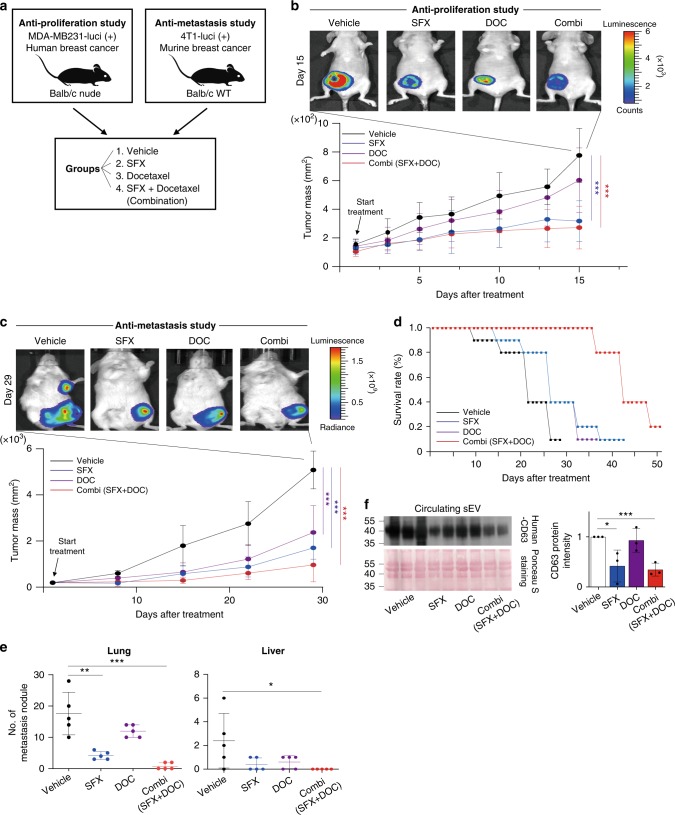
SFX suppresses breast cancer cell proliferation and metastasis in two cancer xenograft models. **a** Schematic illustration of the in vivo experimental designs with different treatments. **b** Top, Representative image of cancer cells tracked with the IVIS imaging system following the injection of mice with luciferase-expressing MDA-MB231 cells. Bottom, Tumor mass volume of in BALB/c *nude* female mice inoculated with MDA-MB231-luci (+) cells and then treated with an indicated drug or vehicle. *n* *=* 7 per group. **c** Top, in vivo images of 4T1 cancer cell-bearing mice using an IVIS imaging system. Bottom, tumor mass volume of 4T1-luci (+) cells inoculated BALB/c *nude* female mice treated with an indicated drug or vehicle *n* *=* 9 per group. **d** Measurement of the survival rates in tumor-bearing mice after different treatments. The survival curves were generated according to the Kaplan–Meier method *n* *=* 5 per group. **e** Quantitative representation of lung and liver nodules from mice bearing 4T1-luci (+) cells. *n* *=* 5 per group. **f** Left top, immunoblot of human CD63 in circulating cancer-cell-derived sEV from MDA-MB231-luci (+)-bearing mice. *n* *=* 3 per group where similar amounts of protein/lane were verified by Ponceau S staining (Bottom). Right, Analysis of the relative protein intensity of CD63 measured using a densitometer system. Significance was determined using an unpaired two-tailed Student’s *t*-test. ***p < 0.001, **p < 0.005, and *p < 0.05. Error bar, SD. Source data (**b**–**f**) are provided as a Source Data file

We also examined the anti-cancer effect of SFX on breast cancer cells because cancer-cell-derived sEV are known to play important roles in cancer progression and metastasis. SFX significantly halted cellular proliferation, colony formation, and cancer cell invasion/migration activities (Supplementary Fig. 5a–e), without obvious cytotoxicity (Supplementary Fig. 3c), compared with the control cells. Based on these in vitro data, we examined the anti-cancer effect of SFX by using two different mouse cancer xenograft models (Fig. 2a). In our previous pharmacokinetic study^21^, SFX showed excellent oral bioavailability and maintained desirable exposure levels at 200 mg kg^−1^ day^−1^ in mice after an oral administration. Before the validation of chemotherapeutic effect of SFX, we studied the subacute oral toxicity profile of SFX. Any pathological signs, including abnormal behaviors, body weight changes, and unexpected death, were evaluated after daily oral administrations of SFX (100, 300, and 900 mg kg^−1^ day^−1^ for 28 consecutive days. Our subacute toxicity study revealed that mice appeared healthy and statistically significant differences in the body weights and various parameters of serum chemistry were not observed between SFX-treated mice and controls, suggesting that SFX up to 900 mg kg^−1^ daily administrations did not cause any obvious clinical indications in both males and females (Supplementary Fig. 6a, b).

Therefore, we evaluated the effect of SFX on the growth rates of MDA-MB231 cells orthotopically implanted into female nude mice. Docetaxel (DOC), currently used intravenously as an anti-cancer agent, was used for control and combination therapy experiments. The growth of MDA-MB231 cells was significantly suppressed by oral administrations of SFX, compared to the vehicle-control group (Fig. 2b). SFX also effectively delayed the metastasis of mouse 4T1 breast cancer xenografts on day 29 relative to the vehicle group (Fig. 2c), thus survival rates were significantly increased (Fig. 2d). In addition, SFX treatment markedly reduced the countable colonies of metastatic foci of 4T1 cancer cells in the lung and liver (Fig. 2e) with massive growth of tumor nodules of the experimental mice, compared with those of control animals. To further study whether SFX can inhibit the secretion of sEV from transplanted cancer cells in vivo, we determined the amounts of circulating human cancer cells-derived sEV in the sera of host mice by using the specific antibody recognizing the human CD63 but not the mouse CD63 (ref. ^12^). Densitometric analysis revealed that the expression of human CD63 exosomal protein was significantly decreased in the SFX-treated animals and DOC-combination group compared to vehicle-exposed controls (Fig. 2f). To further validate whether the anti-cancer effect of SFX is mediated through the inhibition of sEV secretion, we performed rescue experiments using two animal models. The anti-proliferation and anti-metastasis effects of SFX and DOC combination groups were significantly reduced by sEV treatment, suggesting that the anti-cancer effects of SFX are mostly mediated through the inhibition of sEV secretion (Supplementary Fig. 7a–e).

### SFX influences ESCRT-dependent multivesicular endosome biogenesis and secretion

**Fig. 3:**
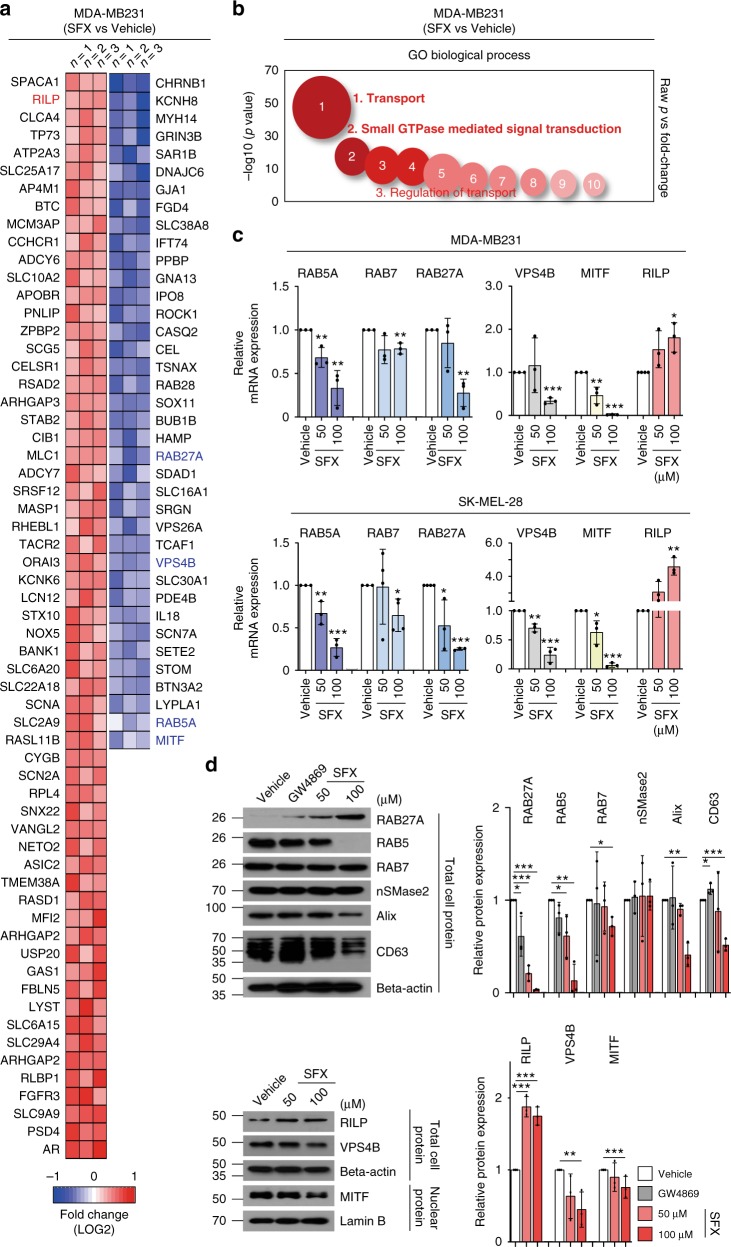
SFX impairs sEV secretion through interfering with the ESCRT-dependent MVE biogenesis. **a** Heatmap of the selected transcriptome of SFX-treated MDA-MB231 cells compared to untreated control cells. Heatmap represents probe sets for transcripts expressed at significantly higher or lower levels after exposure to 100 μM SFX, respectively (FC = ±1.3 *p* value = 0.05). *n* = 3. **b** Advanced bubble chart shows enrichment of differentially expressed genes in the indicated signaling pathways. The *x*-axis label indicates gene ontology (GO) biological processes, and the *y*-axis label represents *p* value. **c** qRT-PCR analysis of various genes in 24 h SFX-treated MDA-MB231 (upper) and 6 h SFX-treated SK-MEL-28 human melanoma cells (lower). *n* = 3. **d** Immunoblot for the indicated proteins in MDA-MB231 cells. Beta-actin and Lamin B were used as loading controls for cell lysates and nuclear fraction, respectively. Right, Analysis of the relative protein intensity of proteins measured using a densitometer system. *n* = 3. Experiments were performed with 95% confluent cells. Significance was determined using an unpaired two-tailed Student’s *t*-test. ***p < 0.001, **p < 0.005 and *p < 0.05. Error bar, SD. Source data (**c**, **d**) are provided as a Source Data file

To investigate whether SFX inhibits sEV secretion through altered expression of the genes involved in sEV biogenesis, we performed a microarray analysis of mRNAs obtained from MDA-MB231 and SK-MEL-28 cells after SFX treatment. Strikingly, most genes down-regulated by SFX treatment in both cell lines encode the regulatory proteins associated with transport and small GTPase-mediated signal transduction (Fig. 3a, b and Supplementary Fig. 8a–c). This result strongly suggests that an upstream factor (a potentially newly-recognized off-target of SFX), which regulates the transcriptions of various genes associated with sEV biogenesis and secretion, is likely present in these cancer cells and that this upstream factor could be regulated by SFX, even though this drug was originally developed as an antibiotic to kill bacteria.

Several RABs (RAB5, RAB7, and RAB27A), VPS4B, and MITF genes were down-regulated by SFX treatment, as confirmed by both quantitative reverse transcription polymerase chain reaction (qRT-PCR) (Fig. 3c) and western blotting (Fig. 3d). Greater amounts of RAB5 and RAB27A proteins were expressed in cancer cells, compared to normal cells (Supplementary Fig. 9a). Moreover, the expressed amounts of RAB5 and RAB27A in breast cancer cells (Fig. 3d) and SK-MEL-28 melanoma cells (Supplementary Fig. 9b) were significantly reduced after treatment with SFX in a dose-dependent manner. The ESCRT machinery is important in the multivesicular endosomes (MVE) maturation^22^. VPS4B^23^, an important ESCRT-related component to regulate intraluminal vesicles formation, and Alix^24^, an ESCRTIII-binding partner, were decreased by SFX (Fig. 3d). Moreover, cellular CD63, ESCRT-dependent or -independent sEV biogenesis regulator^25^, was also down-regulated (Fig. 3d). However, neutral sphingomyelinase (nSmase) enzyme activity, which is related to ceramide-regulated events and a target of GW4869 (ref. ^26^), was not affected by SFX (Fig. 3d and Supplementary Fig. 9c). In addition, SFX significantly suppressed the levels of a transcription factor MITF (Fig. 3d), which can increase the expression of late endosomal proteins, such as RAB7 and CD63, and a main sEV secretion regulator, RAB27a, in melanoma cells^27^. Hence, the decreased levels of RAB7, CD63, and RAB27a might be due to the down-regulation of MITF by SFX. RAB-interacting lysosomal protein (RILP), which is the RAB7 effector required for transport to lysosomes, was markedly upregulated by SFX in a dose-dependent manner (Fig. 3c, d). SFX did not alter the intracellular calcium concentration when compared to dimethyl amiloride (DMA), an inhibitor of the secretion of exosome via reducing intracellular calcium levels, although sEV secretion can be regulated by a calcium-dependent mechanism^28^ (Supplementary Fig. 9d). These results suggest that SFX would influence both sEV biogenesis and secretion through the ESCRT-dependent mechanism.

### SFX inhibits sEV secretion via ETA

**Fig. 4:**
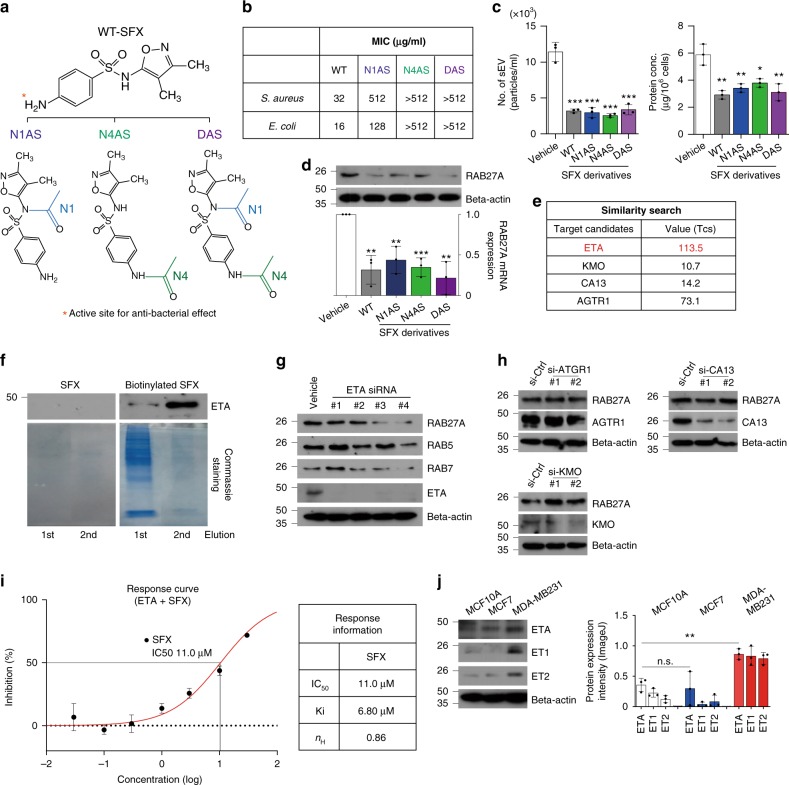
Identification of the endothelin receptor as the novel target of SFX. **a** Schematic illustration of three acetylated SFX derivatives. N1, N1-acetylation site (blue). N4, N4-acetylation site (green). **b** The minimal inhibitory concentration (MIC) of SFX and its derivatives against *Staphylococcus aureus* ATCC 29213 and *Escherichia coli* ATCC 25922. **c** Measurement of the number of sEV and the amounts of sEV proteins. Left, the number of sEV. Right, the amounts of sEV proteins. *n* = 3. **d** Immunoblot (Top) and qRT-PCR analysis (bottom) of RAB27A in MDA-MB231 cells treated with 100 µM SFX or its structural derivatives. **e** Candidate proteins for binding to SFX by searching binding DB. Tanimato coefficients (Tcs) as the measure of similarity are summarized. **f** Detection of ETA by immunoblot. Eluted parts from the membrane fraction of MDA-MB231 cells were used for biotin-based pull-down assay. WT-SFX was used as a negative control. *n* = 3. **g** Immunoblot of various proteins in MDA-MB231 cells after transfection of different ETA siRNAs. AccuTarget^TM^ siRNA was used for control siRNA. *n* = 3. **h** Immunoblot of Rab27a protein after transfection of each siRNA specific to AGTR1, CA13, or KMO into MDA-MB231. *n* = 3. **i** Dose response curve for the inhibition of binding of ^125^I-ET1 to the human ETA receptor. IC_50_, the half maximal inhibitory concentration. Ki the inhibitory constant, *n*_H_ Hill coefficient. **j** Immunoblot of ETA and its ligand, ET1 and ET2 in MCF10A, MCF7, and MDA-MB231 cells. *n* *=* 3 per cell line. Experiments were performed with 95% confluent cells. Significance was determined using an unpaired two-tailed Student’s *t*-test. ***p < 0.001, **p < 0.005 and *p < 0.05. Error bar, SD. Source data (**b**–**d**, **i**–**j**) are provided as a Source Data file

Our data described above strongly indicate that a newly-recognized off-target of SFX exists in cancer cells to exhibit its inhibitory effect on the secretion of sEV. First, to determine whether the active site (the NH_2_-group on the N4 position) of SFX as an antibiotic entity is essential for the inhibition of sEV secretion, three SFX derivatives consisting of two mono-acetylated (N1-acetylated SFX, N1AS and N4-acetylated SFX, N4AS) and one diacetylated SFX (DAS) by modifying the two nitrogen atoms on SFX were synthesized (Fig. 4a). As expected, an antibiotic effect completely disappeared in two derivatives (N4AS and DAS) in which the active site (N4) was blocked by acetylation, as indicated by the minimum inhibitory concentration (MIC) against two microorganisms (MIC >512 in both *Staphylococcus* *aureus* and *Escherichia* coli) (Fig. 4b). Surprisingly, however, all three derivatives still reduced the sEV secretion (Fig. 4c) and the expression of Rab27a in MDA-MB231 (Fig. 4d), similar to the changes induced by SFX. These results strongly suggest that the NH_2_ group at the N4 site is not critical for inhibition of sEV secretion although this group is essential for antibiotic function.

To identify a direct protein target of SFX in cancer cells, we applied an in silico approach based on a Similarity Ensemble Approach (SEA), a method developed by our group^29^. Using data from the BindingDB, SEA identified four proteins (Fig. 4e) as target candidates, which are: endothelin receptor type A (ETA), kynurenine 3-monooxygenase (KMO), carbonic anhydrase 13 (CA13), and angiotensin II type 1a receptor (AGTR1). To determine whether each candidate protein can bind directly to SFX, a pull-down assay was conducted using biotinylated SFX with the lysate or membrane fraction of MDA-MB231 cells. Our analysis revealed that only the antibody recognizing ETA showed a signal in elution parts from the membrane fraction but not the lysate (Fig. 4f). Consistent with these results, the expressions of RAB27A, RAB5, and RAB7 proteins were significantly decreased by siRNA-mediated knockdown of the ETA gene (Fig. 4g), but not by suppression of the other three genes, in MDA-MB231 cells (Fig. 4h). To demonstrate direct binding of SFX with ETA, radioactive binding assay was performed and an IC_50_ value of 11.0 μM SFX was subsequently calculated (Fig. 4i). This binding assay result was consistent with an earlier result reported over two decades ago, where SFX could be an ETA selective endothelin antagonist^30^.

It has been reported that the ETA activation by its agonists (endothelin-1, ET1, and endothelin-2, ET2) promotes cancer progression through a network of cellular pathways and interactions with the tumor microenvironment^31^. Consistently, we observed that ETA and its agonists were strongly expressed in highly invasive MDA-MB231, compared to MCF7 and MCF10A cells (Fig. 4j). Small-molecule antagonists of ETA have been developed as anti-cancer drugs^32^; however, to our knowledge, there has been literally no report on the relationship between ETA and sEV secretion.

**Fig. 5:**
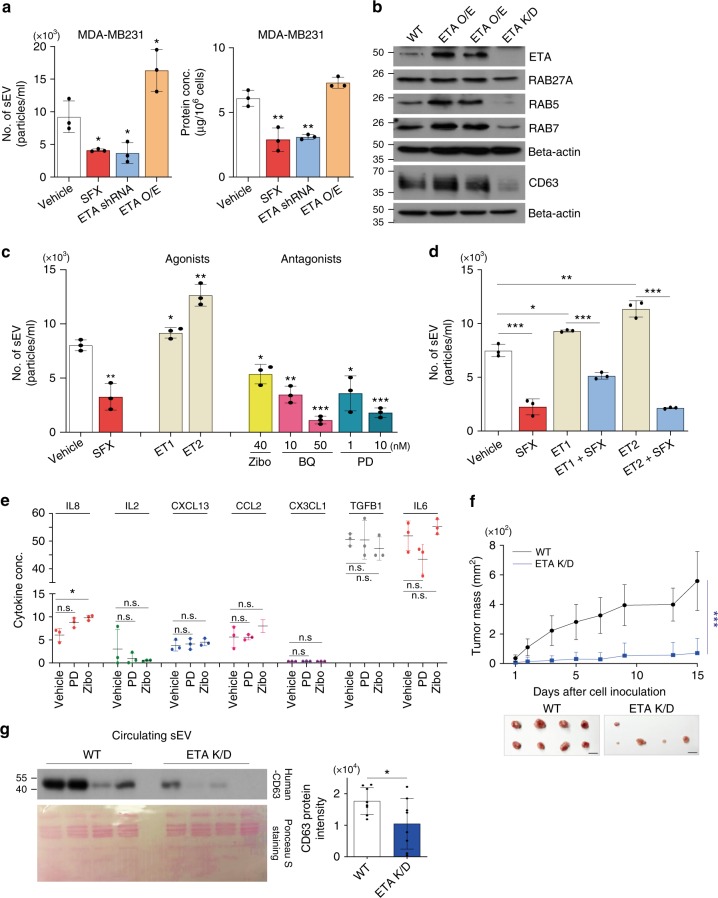
Identification of ETA as the novel regulator of sEV biogenesis. **a** Measurements of the number of sEV secreted (left) and the amounts of sEV proteins (right) from WT, ETA-overexpressed (ETA-OE), and ETA knockdown (K/D) MDA-MB231 cells. *n* = 3. **b** Immunoblot of various proteins in ETA-OE and ETA-K/D MDA-MB231 cells. *n* = 3. **c** Measurement of the number of sEV secreted and the amount of sEV proteins from ETA agonist- or antagonist-treated MDA-MB231 cells. *n* = 3. **d** Measurement of the number of sEV and the amounts of sEV proteins from MDA-MB231 cells treated with 10 nM ET1 or 10 nM ET2 in the presence of 100 μM SFX. *n* = 3. **e** Measurement of soluble proteins secreted from MDA-MB231 cells in the presence of PD156707 or zibotentan for 24 h. The unit of IL8, IL2, CXCL13, CCL2, TGFB1, and IL6 is pg ml^−1^. The unit of CX3CL1 is ng ml^−1^. *n* = 3. **f** Tumor mass volume of in BALB/c *nude* female mice inoculated with MDA-MB231 or MDA-MB231 ETA K/D. Bar, 1 cm. *n* *=* 8 per group. **g** Left top, immunoblot of human CD63 in circulating cancer-cell-derived sEV from MDA-MB231 or MDA-MB231 ETA K/D-bearing mice (*n* *=* 8 per group) where similar amounts of protein/lane were verified by Ponceau S staining (Bottom). Right, densitometric analysis of the relative protein intensity of CD63. Experiments were performed with 95% confluent cells. Significance was determined using an unpaired two-tailed Student’s *t*-test. ***p < 0.001, **p < 0.005 and *p < 0.05. Error bar, SD. Source data (**a**, **c**–**g**) are provided as a Source Data file

We therefore studied the underlying mechanisms by which ETA interferes with sEV secretion. First, to achieve this, MDA-MB231 cells were stably transfected with an ETA shRNA (Knockdown, K/D) or an ETA ORF (over-expression, OE). Similar to SFX treatment, ETA K/D significantly decreased the secretion of sEV (Fig. 5a), with a reduction of RAB27A, RAB5, and RAB7 proteins (Fig. 5b). Conversely, ETA-OE significantly increased the secretion of sEV (Fig. 5a), with elevated levels of RAB5 and RAB7 proteins (Fig. 5b). To further investigate the relationship between ETA and sEV secretion, we studied the effects of several ETA-specific agonists ET1 and ET2 or antagonists (zibotentan, BQ123, and PD159701) on sEV secretion. ET2 significantly increased sEV secretion, which was slightly elevated by ET1, compared to control (Fig. 5c and Supplementary Fig. 10a). In contrast, the three antagonists tested in this study potently inhibited sEV secretion with respect to the number and the protein amounts of secreted sEV (Fig. 5c and Supplementary Fig. 10a). Moreover, the number and the protein amounts of the secreted sEV were still decreased by the combined treatment of SFX and ET1 or ET2 agonist (Fig. 5d and Supplementary Fig. 10b), indicating a potency of SFX in decreasing the sEV secretion despite the presence of ETA agonists. Similar to the SFX-treated cells (Fig. 1e), ETA antagonists did not significantly alter the classical cytokine secretion pathway (Fig. 5e). Consistently, the mRNA expression levels of RAB27a, RAB5, RAB7, RILP, MITF, and VPS4B, as shown in SFX-treated cells (Fig. 3c), were down-regulated by ETA antagonists (Supplementary Fig. 10c). Additionally, we confirmed the relationship between ETA and cancer progression in a breast cancer model. ETA antagonists, zibotentan, BQ123, and PD156707, significantly repressed the invasion and migration activities of cancer cells, MDA-MB231 and 4T1 in vitro (Supplementary Fig. 11a–e). Furthermore, the potent anti-proliferation effects of ETA antagonists through the inhibition of sEV secretion were observed in mice inoculated with MDA-MB231-ETA K/D cells or pretreated with ETA antagonists (Fig. 5f, g and Supplementary Fig. 11a). Taken together, these results suggest that ETA is one of critical elements of the complex machinery for sEV secretion and constitutes an upstream protein for regulating sEV in MDA-MB231 cells.

### ETA antagonists increase fusion of MVE with lysosomes

**Fig. 6:**
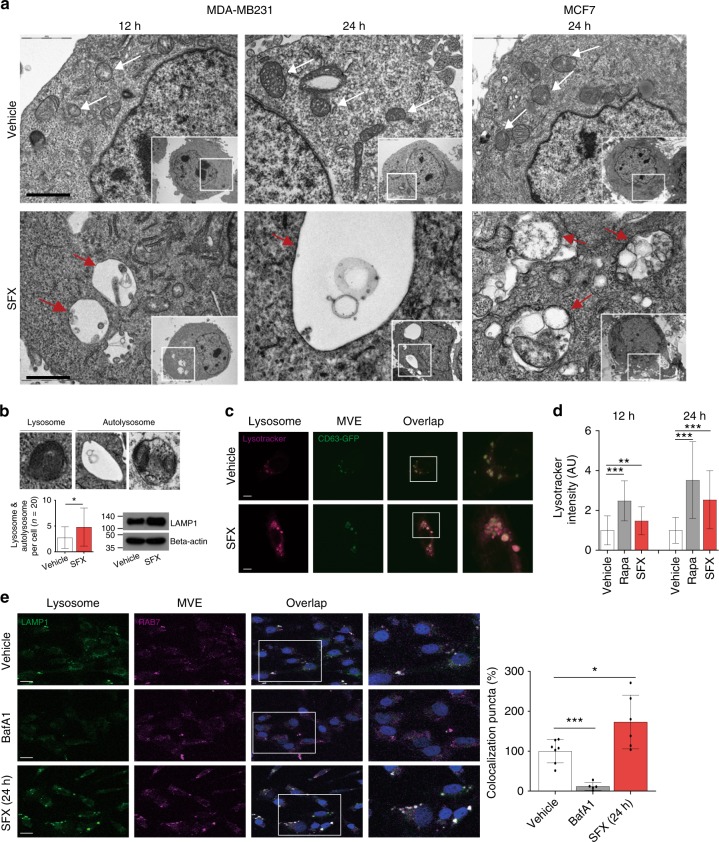
SFX induces fusion of MVE with lysosomes in breast cancer cells. **a** TEM analysis of SFX-treated MDA-MB231 cells. Red arrows indicated unusual structures in SFX-treated MDA-MB231 and MCF7, and white arrows indicated MVEs in control cells. Scale bar, 5 μm. **b** Top, Images of lysosome and autolysosome structures observed in SFX-treated MDA-MB231 cells. Bottom left, the numbers of distributed lysosome and autolysosome structures were counted. *n* = 20. Bottom right, LAMP-1 protein expression was upregulated in SFX-treated MDA-MB231 cells. *n* = 3. **c** Confocal image of CD63-GFP (+)-MDA-MB231 in the presence or absence of SFX. Red, lysotracker. Green, CD63-GFP. Scale bar, 2 μm. **d** Measurement of Lysotracker (red) intensity in rapamycin-treated (positive control) or SFX-treated MDA-MB231 cells. Lysotracker intensity measurement >170 cells per group. **e** Left, Image of MDA-MB231 treated by 100 μM SFX. Green, CellLight-Lysosome-GFP (GFP-LAMP1). Red, CellLight-late endosome/MVE-RFP (RFP-RAB7). Scale bar, 2 μm. Right, Quantitative colocalization rates of lysosomes and MVE in SFX-treated MDA-MB231. Colocalization puncta count >60 cells per group using ImageJ program. Bafilomycin A1 (BafA1) was used as a negative control. Experiments were performed with 95% confluent cells. Significance was determined using an unpaired two-tailed Student’s *t*-test. ***p < 0.001, **p < 0.005 and *p < 0.05. Error bar, SD. Source data (**b**, **d,** **e**) are provided as a Source Data file

To further investigate morphological changes inside cancer cells after SFX treatment, we performed transmission electron microscopy (TEM) analysis of MDA-MB231 cells at 6, 12, and 24 h post-exposure. Interestingly, the structure of degraded autophagic vacuoles (AVs) following lysosomal fusion could be observed 12 h after SFX treatment (Fig. 6a). The most abundant type of vesicles occupying the cytosol of MDA-MB231 cells appeared to be “empty”. In addition, much larger structures occupied extensive areas of the cytosol of MDA-MB231 and MCF7 cells at 24 h after SFX treatment, but the control groups looked normal (Fig. 6a). More importantly, lysosomes or autolysosomes densely filled with multi-lamellar structures were also observed, while the number of these sub-organelle structures and the expression of lysosome-associated membrane proteins 1 (LAMP-1), which is critical in lysosome biogenesis and autophagy^33^, were increased in SFX-treated cells (Fig. 6b).

It has been reported that the balance between autophagy and sEV biogenesis is important for the maintenance of cellular homeostasis^34^. Hence, we investigated whether the relationship between MVE and lysosomes could be affected by SFX. We observed that lysosome activity was strongly increased by SFX (Fig. 6c, d) and the fusion of MVE with lysosomes was accelerated by SFX treatment relative to the control (Fig. 6e). Importantly, these observations have led to the hypothesis that SFX initially influences ESCRT-dependent MVE biogenesis, followed by the imperfect MVEs moving to the lysosomes for degradation.

**Fig. 7:**
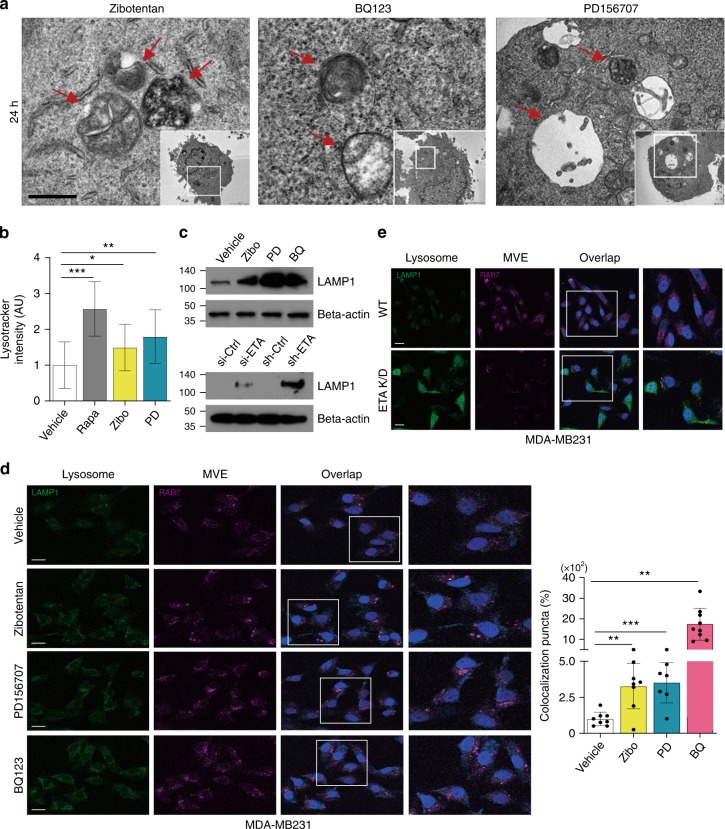
Antagonists against the endothelin receptor induce fusion of MVEs with lysosomes in MDA-MB231 cells. **a** TEM analysis of ETA-antagonist-treated MDA-MB231 cells. Scale bar, 5 μm. **b** Measurement of lysotracker (red) intensity in rapamycin-treated or antagonist (Zibotentan and PD156707)-treated MDA-MB231. Lysotracker intensity measurement >100 cells per group. **c** Top, Immunoblot of LAMP-1 protein in antagonist (Zibo, BQ-123, and PD156707)-treated MDA-MB231. Bottom, Immunoblot of LAMP-1 protein in ETA-siRNA-transfected MDA-MB231 and in ETA-K/D MDA-MB231. *n* = 3. **d** Left, Image of MDA-MB231 treated by SFX. Green, CellLight-Lysosome-GFP (GFP-LAMP1). Red, CellLight-late endosome/MVE-RFP (RFP-RAB7). Scale bar, 2 μm. Right, Quantitative colocalization rates of lysosomes and MVE in MDA-MB231 treated with Zibotentan, PD156707, or BQ123. Colocalization puncta count >65 cells per group. **e** Images of ETA K/D MDA-MB231 or WT MDA-MB231 cells. Different colors represent the same markers as described in **d**. Scale bar, 2 μm. Experiments were performed with 95% confluent cells. Significance was determined using an unpaired two-tailed Student’s *t*-test. ***p < 0.001, **p < 0.005 and *p < 0.05. Error bar, SD. Source data (**b**, **d**) are provided as a Source Data file

Next, we investigated whether the ETA signaling pathway is related to the fusion of lysosomes and MVEs. Similar to the effects in SFX-treated cells, autolysosomes or AVs were detected in antagonist-treated MDA-MB231 cells (Fig. 7a). Moreover, confocal microscopy verified co-localization of MVEs with lysosomes, the increase in lysosomal activity, and the up-regulation of LAMP-1 in antagonist-treated or ETA-siRNA-transfected MDA-MB231 cells (Fig. 7b–e). These results strongly suggest that one of the major targets of SFX in MDA-MB231 cells is ETA, which could be a GPCR protein newly-recognized for the regulation of sEV biogenesis and secretion.

## Discussion

Breast cancer is the most prevalent cancer in women and the leading cause of death worldwide, up to half-a-million deaths annually, despite surgical treatments combined with advanced radiotherapy and chemotherapy. Metastasis is a major cause for deaths of many patients with various types of cancer, including breast cancer. Metastatic dissemination of breast cancer can occur in a late stage of cancer progression^35^ as well as in preinvasive stages of tumor progression, by various systemic factors secreted from the tumors^36^. The exosomes and/or sEV, emerging players of systemic factors, play an important role in establishing premetastatic niche in future metastatic organs and furthermore in dictating organotrophic metastasis by integrin repertoire of sEV in breast cancer cells^37^. Therefore, early detection as well as prevention of metastasis by conventional treatments and/or sEV secretion could become very important in reducing the cancer-related pathologies and deaths.

Despite significant advancement in exosome/sEV research in recent decades, no drugs targeting to inhibition of sEV secretion have been approved for human usage. In this study, we identified SFX as an inhibitor of the secretion of sEV by high-throughput screening of a library of FDA-approved drugs. SFX affects the expression of many genes associated with the pathways of sEV biogenesis and secretion in cancer cells (Fig. 3). Our results suggest that an upstream target, as a newly-recognized off-target, of this old drug might regulate the sEV-associated pathways in breast cancer cells. From our computational approach, ETA was selected as the prime target of SFX with the highest priority and was confirmed by a direct binding study, genetic and pharmacology approaches. Therefore, we showed that SFX exhibits a potential anti-cancer effect that is mediated through ETA dependent-inhibition of sEV biogenesis/secretion.

For physiologically relevance, we simply compared the concentration of SFX as an inhibitor of sEV secretion with that of SFX as an antibiotic in vitro and in vivo. As shown in Supplementary Fig. 11, each MIC of SFX was 16 and 32 μg ml^−1^ in *E. coli* and *S. aureus* in this study, converted to 60 and 120 μM, respectively, and thus the concentration (up to 100 μM in this study) for inhibition of sEV secretion in cancer cells could be reasonable. Furthermore, to evaluate whether the dose for mouse study can be physiologically relevant in humans, we used a formula for dose translation from animal to human studies^38^. The dose (200 mg kg^−1^ day^−1^) used in this study was about 4.1–8.2 times lower than the maximal dosage as an antibiotic in human usage. Therefore, we believe that the concentrations of SFX used in this study, as an inhibitor of sEV secretion in cancer cells and as an anti-cancer agent in two different mouse models of breast cancer xenografts, could be physiologically relevant.

Gut microbiota is a critical factor for many pathophysiological conditions such as immune reaction and inflammatory processes^39^. SFX, an antibiotic, may decrease gut microbiome density and/or modify its composition. Several bacteria phyla are shown to critically important in cancer development during dysbiosis^40,41^. Based on the recent reports, gut microbiota is considered as a holistic hub point for cancer development, thus antibiotics-mediated microbiome modulation can be a novel anti-cancer strategy^42,43^. Antibiotics-mediated gut microbiome changes might be partially responsible for the anti-cancer effect of SFX. However, antagonists (not antibiotics) to ETA, a target of SFX, also showed the anti-proliferation effect through the inhibition of sEV secretion (Supplementary Fig. 5a). Therefore, these results suggested that the inhibitory effect of SFX on cancer cell proliferation and metastasis mostly depends on the inhibition of sEV although its effect may come from other properties.

Recent reports suggested that the stimulation of GPCR by its agonists initiates the signaling cascade to regulate exosome formation and secretion. For instance, the activation of GPR143 by its ligand, l-DOPA, halted the secretion of exosomes for intercellular communication in the eye^44^. Activated group I mGluRs increase the secretion of exosomes by calcium release from the endoplasmic reticulum via the secondary messenger, IP3^45^. The activation of histamine H1 receptor in HeLa cells also increases the secretion of exosomes through the promotion of MVB-PM fusion^46^. Some GPCRs, such as A_2A_ receptors, can be transferred via exosomes from the source to the recipient target cells^47^.

In this study, we first demonstrated that ETA is associated with sEV biogenesis and secretion in breast cancer cells. The endothelin receptors (ETRs), which are Family A (Class 1) GPCRs, consist of two receptor subtypes, ETA and ETB. In particular, the activation of the ETA pathway, by ET1 or ET2, induces various effects in cancer cells, such as growth, metastasis, and angiogenesis^32,48^. Thus, ETRs have emerged as key targets for cancer therapy, and small-molecule antagonists, including zibotentan and BQ123, have been tested in humans for cancer drug development^49^.

The increased degradation of MVEs via the autophagy–lysosome pathway was demonstrated by ultrastructural analysis when breast cancer cells were treated with SFX or specific ETA antagonists (Figs. 6a and 7a). Another report showed that ETA is related with autophagy regulation in H9C2 myoblasts^50^. Therefore, we additionally studied whether the inhibitory effect of SFX and ETA antagonists depend on the autophagy pathway, even if the elevation of autophagosome structures was not clearly observed in cancer cells by treatment of these drugs. Through the expression of LC-3BII protein, one of the critical markers in macroautophagy pathway, and the additional LC3B puncta study, SFX and ETA antagonists did not fully induce the LC3B-dependent autophagy pathway. Furthermore, these drugs still inhibited sEV release in LC3B K/D cells. These results suggested that the degradation of MVEs by SFX or ETA antagonists might not be related to the LC3B-dependent autophagy pathway. However, we could not exclude the possibility that other non-canonical autophagy pathways might function partially in cancer cells following treatment with ETA antagonists. Therefore, further work is required to identify the detailed mechanism of the degradation pathway via ETA antagonists for the reduction of sEV biogenesis and secretion.

**Fig. 8:**
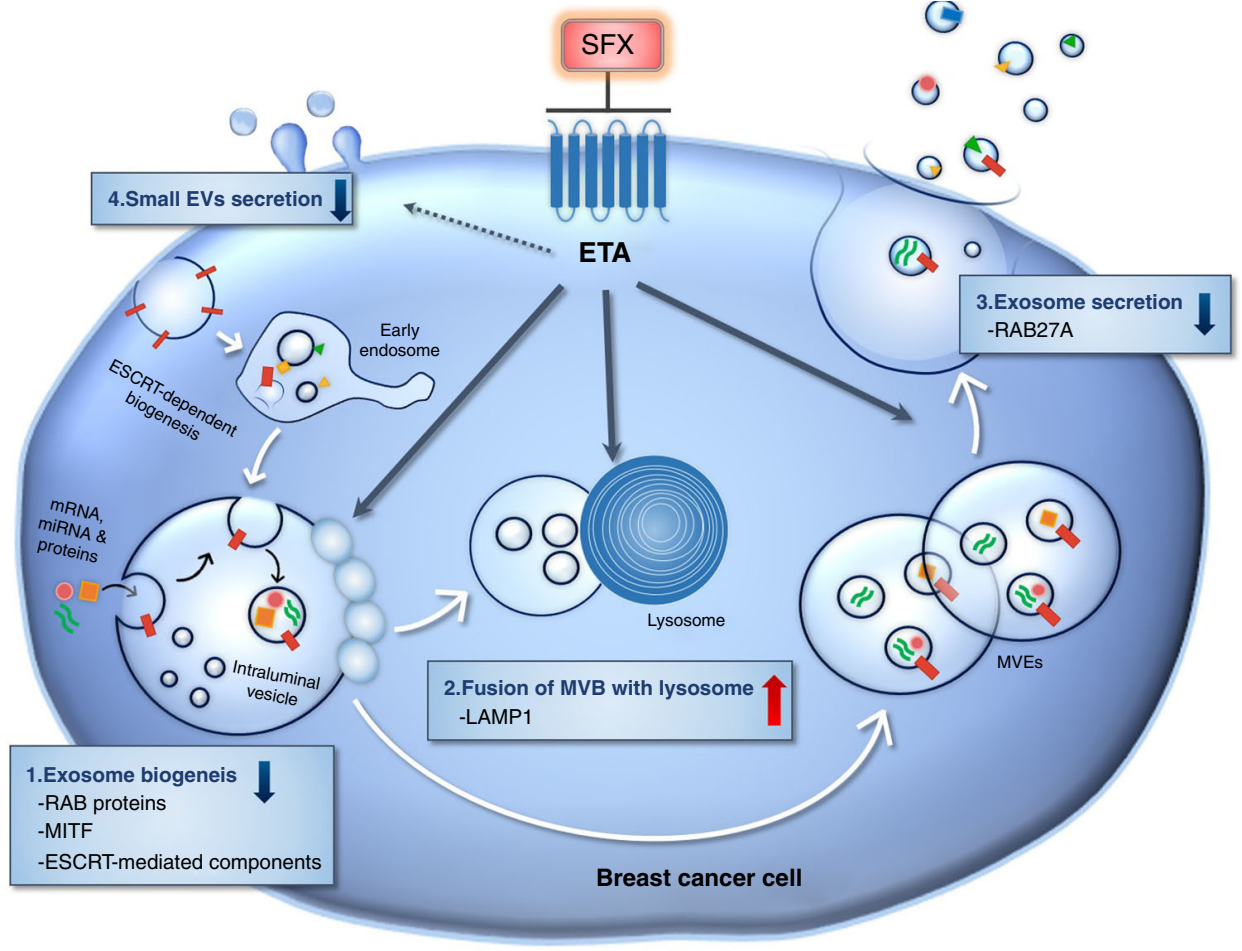
Proposed model for SFX-mediated inhibition of sEV secretion in MDA-MB231 cancer cells. SFX, through ETA binding and subsequent interference, reduced the expression of proteins involved in the sEV biogenesis and secretion, increased the fusion of MVEs with lysosomes, and finally decreased the number of secreted sEV from breast cancer cells

Here, we found that ETA, selected as a newly-recognized off-target of SFX, is associated with sEV biogenesis and secretion, and this finding will accelerate the development of a novel class of drugs through mechanistic studies on the regulation of sEV biogenesis or secretion (Fig. 8). Furthermore, SFX and ETA antagonists can interfere with the ETA function, suppress the secretion of sEV, and change the components of sEV cargo from cancer cells, contributing to anti-cancer effects. Therefore, these ETA-related SFX results are novel and very important for clinical implications although we only tested the effects of SFX and ETA antagonists in breast cancer cells. We expect that these compounds can also beneficially affect other types of cancer cells possibly through preventing sEV biogenesis and secretion, although this needs to be confirmed by future experiments.

## Methods

### Cells and cell culture

All breast cancer and other cells were obtained from the American Type Culture Collection (ATCC) and grown at 37 °C under humidified atmosphere with 5% CO_2_ and 95% air using the recommended culture medium. MCF7, MDA-MB231, and HEK293T cells were cultured in Dulbecco’s modified Eagle’s medium (Hyclone) with 10% fetal bovine serum (FBS) and 1% antibiotics. MCF10A cells were grown in mammary epithelial cell growth medium (MEGM, Lonza) with 5% FBS, 52 μg ml^−1^ bovine pituitary extract, 0.5 μg ml^−1^ hydrocortisone, 10 ng ml^−1^ EGF, and 5 μg ml^−1^ insulin. SK-MEL-28 cells were cultured in Minimum Essential Medium with Earle’s Balanced Salts (MEM/EBSS; Hyclone). For drug treatment experiments, cells were washed and incubated with FBS-free medium after cell cultures reached 90% confluency, except invasion, migration, colony formation, and proliferation assays.

### Chemicals

An FDA-approved drug library was purchased from Selleckchem (L1300) and used for in vitro screening study. The following chemicals were purchased from Sigma Aldrich, including Sulfisoxazole (SFX, S6377), 5-(*N*,*N*-dimethyl)amiloride hydrochloride (DMA, A4562), GW4869 (D1692), Rapamycin (R8781), FTY720 (SML0700), ET1 (E7764), BQ123 (B150), PD156707 (PZ0141), zibotentan (SML1550), and Bafilomycin (B1793). ET2 (#1164) was obtained from Tocris Bioscience. Docetaxel (DOC) was a gift from professor Keon-Wook Kang (Seoul National University).

### Fluorescence-based high-throughput screening assay

MDA-MB231 cells were transfected with pCT-CD63-GFP (pCMV, Exosome/Secretory, CD63 Tetraspanin tag) plasmid (System Bioscience) to construct MDA-MB231-CD63-GFP stable cell line. For high-throughput screening assay, MDA-MB231-CD63-GFP (+) cells (1 × 10^4^/well) were seeded in 96-well culture plates and grown overnight. These cells were subsequently incubated with 1163 individual FDA-approved drugs (Selleckchem) for 24 h under serum-depleted condition. The culture supernatants were then transferred into 96-well black plates and fluorescence from each supernatant was measured at 485 nm (excitation) and 538 nm (emission) using a fluorescence microplate reader (GeminiEM; Molecular Devices, San Jose, CA).

### FACS

For green fluorescence protein (GFP) detection, cells were re-suspended in FACS buffer (phosphate-buffered saline (PBS) with 5% fetal calf serum). The GFP intensities in MDA-MB231 cells were determined by FACSCalibur (BD Bioscience).

### Proliferation and cytotoxicity assay

Concentration- and time-dependent effects on cell proliferation and cytotoxicity were measured using the MTT [3-(4,5-dimethylthiazol-2-yl)−2,5-diphenyltetrazolium bromide] assay. In the cytotoxicity assay, cells were seeded in a 24-well plate at a density of 2 × 10^4^ cells/well and grown for 24 h. Then, the culture medium was replaced with a fresh medium containing 2% FBS and different concentrations of SFX for another 24 h. At the end of the incubation, 500 μg ml^−1^ MTT reagent was added to each well and incubated for 3 h at 37 °C. After removal of the MTT containing supernatant solution, 100 μl dimethyl sulfoxide (DMSO) was added to each well to dissolve violet formazan crystals and optical density of each well was assessed with a microplate reader at 550 and 570 nm. The proliferation assay followed the same procedure, except that the low confluency (starting with 8%) of cells were treated with SFX every 24 h to measure the change in proliferation over time.

### Isolation of MVs and sEV

The MVs were isolated by following the method reported by Rong Xu. et al.^51^. Briefly, the individual supernatants from MCF7, MDA-MB231, and SK-MEL-28 cells were serially centrifuged at 300 × *g*/3 min and 2500 × *g*/20 min. Then, the supernatants were filtrated using a 0.8 μm syringe filter and centrifuged at 10,000 × *g*/40 min again. The MV pellets were re-suspended with PBS and examined with the nanoparticle tracking analysis (NTA). To isolate sEV, individual supernatants obtained from the indicated cells were serially centrifuged at 300 × *g*/3 min, 2500 × *g*/20 min, and 10,000 × *g*/30 min. Then the supernatants were filtrated using a 0.2 μm syringe filter, and centrifuged at 120,000 × *g*/90 min. The sEV pellets were re-suspended with PBS and centrifuged at 120,000 × *g*/90 min again. The purified sEV pellets were re-suspended with PBS or 1× RIPA buffer for further experiments.

For miRNA microarray assay, ExoQuick-TC exosome precipitation solution (System Bioscience) was used for sEV isolation with the method optimized by our group^52^. Briefly, MDA-MB231 cells were treated with vehicle or SFX in FBS-free media, and incubated for 24 h. Next, conditioned media was sequentially centrifuged at 300 × *g*/3 min, 2500 × *g*/20 min, and 10,000 × *g*/30 min, and then conditioned media were filtrated using 0.2 μm filter. These medium were incubated with ExoQuick-TC solution at 4 °C overnight. After incubation, sEV were isolated by centrifugation at 1500 × *g*/30 min and washed with PBS three times. Finally, sEVs were re-suspended in nuclease-free water (Promega) for total RNA extraction.

### Nanoparticle tracking analysis

Cell culture supernatants containing MVs and sEV were analyzed using a NanoSight LM10 device (Nanosight, Malvern). A monochromatic laser beam at 405 nm was set to analyze the nanoparticles, and a video with a 30-s duration was taken at a rate of 30 frames/s and a camera level of 7. Approximately 30–100 particles were analyzed in each field of view, and then particle brown-movement was assessed using NTA software (version 2.3, Nanosight). NTA post-acquisition settings were optimized and kept constant between samples, and recorded video was then analyzed to measure particle sizes and concentrations.

### Determination of sEV protein concentration

Concentration of sEV proteins was determined using the microBCA assay (#23235; ThermoFisher). To measure protein concentrations, sEV were isolated by differential ultracentrifugation. sEV proteins were extracted using 1× RIPA buffer (#50–188; Merch Millipore). The microBCA assay was performed according to the manufacturer’s instructions.

### ELISA

The levels of IL8, IL2, IL6, CX3CL1, CXCL13, CCL2, and TGFB1 cytokines/chemokines were measured by sandwich enzyme-linked immunoassay (ELISA). Briefly, MDA-MB231 cells (3 × 10^5^/well) were seeded in six-well plates overnight, and treated with SFX, PD156707, or BQ123 for additional 24 h. Subsequently, the supernatants were centrifuged at 300 × *g*/3 min to remove the debris and used for the ELISA. For the measurement of cytokine, the following ELISA kits from R&D systems were used: IL8 (D8000C), IL2 (D2050), IL6 (D6050), CX3CL1 (DCX310), CXCL13 (DCX130), CCL2 (DCP00), and TGFB1 (DB100B).

### RNA extraction and qRT-PCR

Total RNA from different cells was extracted by using TRIzol reagent (#15596026; Invitrogen) according to the manufacturer’s recommendation. A total of 2 μg RNA was reverse-transcribed using the RT-premix (K2041; Bioneer). qRT-PCR gene expression analysis was performed in three biological replicates using gene-specific qRT-PCR oligonucleotides. RT-PCR reactions were monitored on an ABI stepOne Plus instrument (Applied Biosystems) using the SYBR premix (#4368577; ThermoFisher). Each sample was PCR-amplified from the same amount of cDNA template. Following an initial step in the thermal cycler for 15 min at 95 °C, the PCR amplification was proceeded for 40 cycles of 15 s at 95 °C and 1 min at 60 °C, and completed by melting curve analysis to confirm specificity of the PCR products. The baseline and threshold values were adjusted according to the manufacturer’s instructions. Primer sequences used for RT-PCR (5′ to 3′) are summarized in Supplementary Table 1.

### Microarray analysis

The Affymetrix Whole-Transcript Expression Array process was executed according to the manufacturer′s recommended protocol (GeneChip Whole Transcript PLUS reagent kit). cDNA was synthesized using a GeneChip WT (Whole Transcript) amplification kit according to the manufacturer’s instructions. The sense cDNA was fragmented and biotin-labeled with TdT (terminal deoxynucleotidyl transferase) using a GeneChip WT Terminal Labeling kit. Approximately 5.5 μg of labeled DNA target was hybridized to the Affymetrix GeneChip Clariom S Human Array at 45 °C for 16 h. The hybridized arrays were washed, stained on a GeneChip Fluidics Station 450, and scanned on a GCS3000 Scanner (Affymetrix). Signal values were computed using Affymetrix® GeneChip™ Command Console software. The data were summarized and normalized using the robust multi-average (RMA) method implemented in Affymetrix® Power Tools (APT). Gene-enrichment and functional annotation analysis for the significant probe list was performed using Gene Ontology (http://geneontology.org) and KEGG (http://kegg.jp) technology. For sEV miRNA microarray, the Affymetrix miRNA Expression Array process was executed according to the manufacturer’s recommended protocol (miRNA 4.0 chip). Raw data were extracted automatically in Affymetrix data extraction protocol using the software provided by Affymetrix GeneChip® Command Console® Software (AGCC). The CEL files import, miRNA level RMA + DABG-All analysis, and result export were performed by using Affymetrix® Power Tools (APT) Software.

### Proteomics

sEV proteins were digested by trypsin^52^ and the tryptic peptides were analyzed by nano-ultra-high-performance LC (UPLC) (Waters) and tandem mass spectrometry using a Q-Tof Premier (Waters)^53,54^. Digested peptides were injected into a 2 cm × 180 μm trap column and resolved in a 25 cm × 75 μm nanoACQUITY C18 column (Waters) using the LC system. All samples were analyzed in triplicate. For protein identification, MS raw data were converted into peak lists using MASCOT Distiller version 2.1 (Matrix Science, London, UK) with default settings. All MS/MS raw data were analyzed using MASCOT version 2.2.1 (Matrix Science)^53^. Mascot was used to search the SwissProt database (release 2018_07) with human taxonomy. Quantification was performed using PEAKS Studio version 10.0 (Bioinformatics Solution Inc., Waterloo, Canada). For Label-free protein quantification, identified peptides were filtered based on False Discovery rate <1%. The abundance of each peptide was determined using ion chromatography extraction and the protein ratio was calculated using the average abundance among the corresponding peptides. Protein ratios were considered acceptable when the proteins contained more than one unique peptide.

### Western blotting

Cellular or sEV proteins were resolved by sodium dodecyl sulfate polyacrylamide gel electrophoresis, transferred onto nitrocellulose membranes, probed with the respective primary antibody, and incubated with a horseradish peroxidase-linked secondary antibody. Images were visualized using enhanced chemiluminescence (ECL) detection reagents (#34580; Thermo Scientific) and quantified using ECL hyper-film (AGFA; Morstel).

The following primary antibodies were used: anti-CD63 (ab68418, 1:1000; Abcam), CD9 (ab2215, 1:1000; Abcam), CD81 (ab109201, 1:1000; Abcam), Alix (ab56932, 1:1000; Abcam), Tsg101 (ab30871, 1:1000; Abcam), Filotillin-1 (#3253, 1:1000; CST), LAMP-1 (ab25630, 1:1000; Abcam), RILP (ab128616, 1:1000; Abcam), beta-actin (4670, 1:3000; Cell Signaling Technology (CST)), Rab27a (ab55667, 1:1000; Abcam), Rab5 (ab18211, 1:1000; Abcam), Rab7 (ab50533, 1:1000; Abcam), EDIL3 (ab88667, 1:1000; Abcam), Glypican-1 (PA5-28055, 1:1000; ThermoFisher), HSP90a (#8165, 1:1000; CST), ASMase (#3687, 1:1000; CST), nSMase2 (ab68735, 1:1000; Abcam), LC3B (NB600-1384, 1:1000; Novus Biologicals), Endothelin receptor type A (ab117521, 1:2500; Abcam), Angiotensin II type I receptor (ab18801, 1:1000; Abcam), Carbonic anhydrases-13 (ab135986, 1:1000; Abcam), Kynurenine 3-monooxygenase (ab130959, 1:1000; Abcam), Endothelin-1 (ab2786, 1:1000; Abcam), Endothelin-2 (sc293248, 1:1000; SCBT), VPS4B (ab102687,1:1000; Abcam), MITF (#12590, 1:1000, CST), and Lamin B (#13435, 1:1000; CST). For the detection of specific human cell-derived sEV, the following primary antibodies were used: the anti-human CD63 (SHI-EXO-M02, 1:1000; Cosmo Bio Co., Ltd).

### miRNA expression analysis

sEV miRNA was extracted according to the manufacturer’s instruction (total exosome RNA isolation kit, #4478545; ThermoFisher). Briefly, after ultracentrifugation, the sEV pellets were re-suspended with the exosome resuspension buffer, and then added 2× denaturating solution and Acid-Phenol:Chloroform to separate into aqueous and organic phases. The total RNA-containing aqueous phase was then transferred to filter cartridge for miRNA isolation. Extracted miRNA was washed using the miRNA wash solution and eluted using the elution buffer. TagMan® MicroRNA system was used for miRNA expression analysis. First, RT master mix [extracted miRNA, dNTPs (with dTTP), reverse transcriptase and RNase inhibitor] was loaded into the thermal cycler to perform reverse transcription using following parameter values: 30 min at 16 °C, 30 min at 42 °C, and 5 min at 4 °C. Then, the fluorescent signals of specific miRNAs by real-time PCR system were detected and recorded. The reaction mixture contained: TagMan MicroRNA assay reagent (ThermoFisher), RT product, and TagMan universal PCR master mix (#4304437; ThermoFisher). U6 snRNA was used as reference. miRNA sequence used for miRNA expression analysis is summarized in Supplementary Table 2.

### RNAi analysis

Transient transfection was performed by using Lipofectamine 3000 (L3000015; ThermoFisher) according to the manufacturer’s instructions. Briefly, cells were seeded in 60 mm dishes at 70–80% confluence, and then were transfected with 2.5 μg siRNA with Lipofectamin 3000 under serum-reduced condition. After 1 day, culture media were replaced with a serum-containing medium, and then RNA and proteins were extracted after 48–72 h post-transfection. siRNAs of ETA, AGTR1, KMO, and CA13 were purchased from Dharmacon; negative control siRNA was purchased from Bioneer (AccuTarget^TM^ Negative Control siRNA, SN-1001-CFG). The siRNA sequence used for RNAi interference analysis is summarized in Supplementary Table 3.

### Sphingomyelinase (sMAse) activity measurements

Acidic sphingomyelinase activity (acidic sMAse, ab190554; Abcam) and sphingomyelinase activity (neutral sMAse, ab138877; Abcam): acidic and neutral sphingomyelinase activities were determined by using an assay kit. Briefly, cells were co-cultured with SFX for 24 h and then lysed with 1× mammalian lysis buffer. These samples were then reacted with the acidic sMAse assay reagents according to the manufacturer’s recommended protocol. After the incubation, fluorescence from each sample was obtained by using a microplate reader (GeminiEM; Molecular Devices) at Ex/Em = 540/590 nm (the cut off was 570 nm). The fluorescence in a blank well was used as a negative control; GW4869 and FTY720 were used for positive controls of neutral SMase and acidic SMase, respectively.

### Intracellular calcium concentration measurements

[Ca^2+^]_i_ concentration was measured using fluo-3/AM (F1242; Invitrogen). For the experiments, MDA-MB231, MCF7, and SK-MEL-28 cells were loaded with 5 μM fluo-3/AM for 30 min at 37 °C. Subsequently, the cells were scanned using a fluorimeter (GeminiEM; Molecular Devices) and the collected data were analyzed according to the manufacturer’s instructions.

### Transmission electron microscopy (TEM) analysis

For TEM analyses to evaluate cell morphologies and sub-organelle structures, drug-treated or control MDA-MB231 CD63-GFP fusion cells were pelleted by centrifugation, and pelleted cells were fixed with 2.5% glutaraldehyde in a 0.1 M phosphate buffer. After washing several times with 0.1 M carcodylate buffer, cells were dehydrated by gradient series of ethanol (50%, 60%, 70%, 80%, 90% ethanol for 20 min each step, 100% 20 min twice) followed by propylene oxide for twice. Afterwards, cells were infiltrated with progressively concentration Eponate 812, and then polymerized in fresh Eponate 812 for 2 days at 60 °C. Samples were sectioned using an Ultra microtome (Ultracut UCT; Leica) and stained with uranyl acetate and lead citrate. Sections were examined with energy filtering TEM (LEO-912AB OMEGA; Carl Zeiss) at the Korean Basic Science Institute Chuncheon Center.

For TEM analyses to detect sEV, MDA-MB231 and MCF7 cells were pelleted by serial ultracentrifugation, and the pellets were deposited on pure carbon-coated EM grids. After staining with 1% uranyl acetate, the grids were dried at room temperature and viewed at ×12,000 magnification using a Biotransmission electron microscope (HT7700; Hitachi) operated at 120 kV.

### Immunofluorescence staining and confocal microscopy

Cells were seeded onto glass coverslips at 2 × 10^4^ cells/well in a six-well confocal chamber overnight and were treated with SFX for 24 h. For the detection of lysosomal activity, 500 nM Lysotracker (L7528; ThermoFisher) was added into the culture medium for 1 h before fixing with 4% paraformaldehyde in PBS for 30 min at room temperature. Fixed cells were washed with PBS, and the coverslips were stained and mounted using DAPI-containing mounting solution. For labeling of lysosomes and late endosomes, 1 × 10^5^ cells were transfected with red fluorescence protein (RFP)-RAB7 vector (CellLight Late Endosomes-GFP, BacMam 2.0, C10588; Molecular Probes) and GFP-LAMP-1 vector (CellLight-lysosome-GFP, BacMam 2.0, C10596; Molecular Probes) and grown overnight. Fluorescence images were obtained with a Ziess LSM5 laser scanning fluorescence confocal microscope. Colocalization puncta was measured using ImageJ.

### Radioligand binding assay

Radioligand binding assay was performed in Eurofins Panlab Discovery Service Center (Taiwan). To evaluate the binding activity of SFX, assay was performed under the following conditions. Human recombinant CHO-K1 cells were used as the source, and ligand concentration was 0.030 nM [^125^I] endothelin-1. IC_50_ values were determined by a non-linear, least-squares regression analysis using MathIQTM (ID Business Solutions Ltd, UK). The inhibition constants (Ki) were calculated by the equation of Cheng and Prusoff^55^ using the observed IC_50_ of the tested compound, the concentration of radioligand employed in the assay, and the historical values for the KD of the ligand (obtained experimentally at Eurofins Panlab, Ltd). The Hill coefficient (*n*_H_), defining the slope of the competitive binding curve, was calculated by using MathIQTM.

### Synthesis of a sulfisoxazole-based affinity probe

First, we synthesized 4-((4-(*N*-(3,4-dimethylisoxazol-5-yl)sulfamoyl)phenyl)amino)-4-oxobutanoic acid (SP-1). To a solution of SFX (557 mg, 2.08 mmol), Et_3_N (0.58 mL, 4.16 mmol) and DMAP (127 mg, 1.04 mmol) in CHCl_3_ (4 mL) were added dropwise into methyl 4-chloro-4-oxobutanoate (0.257 mL, 2.08 mmol) at 0 °C. The reaction mixture was then stirred at room temperature for 2 h and washed with saturated aqueous NaHCO_3_ (2 × 50 mL). The reaction mixture was extracted three times with ethyl acetate and the combined organic phase was washed with brine, dried over anhydrous MgSO_4_, and concentrated under reduced pressure. The residue was purified by flash column chromatography on a silica gel (ethyl acetate/*n*-hexane = 1:1) to yield the amide (310 mg, 39%). ^1^H-NMR (600 MHz, CDCl_3_) δ 9.12 (bs, 1H), 7.67 (d, 2H, *J* = 13.2 Hz), 7.53 (d, 2H, *J* = 12.6 Hz), 3.64 (s, 3H), 2.68 (d, 2H, *J* = 7.2 Hz), 2.66 (d, 2H, *J* = 7.2 Hz), 2.01 (s, 3H), 1.65 (s, 3H); HRMS (FAB+): mass calculated for C_16_H_20_N_3_O_6_S [M + H]^+^, 382.0995; found, 382.1072. Additionally, to the solution of the amide compound (105 mg) in tetrahydrofuran (10 mL), lithium hydroxide monohydrate (105 mg) in H_2_O (10 mL) was added at 0 °C. The reaction mixture was stirred for 48 h and concentrated under reduced pressure. The residue was purified by flash column chromatography on a silica gel (MeOH/CH_2_Cl_2_/AcOH = 1:10:0.1) to yield acid (**1**) (81 mg, 80%). ^1^H-NMR (600 MHz, DMSO-d_6_) δ 12.15(bs, 1H), 10.90 (bs, 1H), 10.40 (s, 1H), 7.77 (d, 2H, *J* = 9 Hz), 7.68 (d, 2H, *J* = 8.4 Hz), 2.68 (t, 2H, *J* = 6.6 Hz), 2.54 (t, 2H, *J* = 6.6 Hz), 2.07 (s, 3H), 1.61 (s, 3H); ^13^C-NMR (150 MHz, DMSO-d_6_) δ 174.1, 174.0, 171.4, 161.7, 143.8, 128.3, 119.0, 118.9, 31.6, 29.2, 29.0, 10.8, 6.3; HRMS (FAB): mass calculated for C_15_H_18_N_3_O_6_S [M + H]^+^, 368.0838; found, 368.0913. Next, we synthesized *N*-(17-azido-3,6,9,12,15-pentaoxaheptadecyl)-5-((3aS,4S,6aR)-2-oxohexahydro-1H-thieno[3,4-d]imidazol-4-yl)pentanamide (SP-2). To a solution of biotin-ONp (300 mg, 0.82 mmol) in THF (5 mL), 17-azido-3,6,9,12,15-pentaoxaheptadecan-1-amine (250 μL, 0.82 mmol) and Et_3_N (350 μL, 2.46 mmol) were added at room temperature. After stirring for 12 h at room temperature, the solution was concentrated under reduced pressure. The residue was purified by flash column chromatography on a silica gel (MeOH/CH_2_Cl_2_ = 1:10) to yield the azide (**2**) (401 mg, 92%). ^1^H-NMR (600 MHz, CD_3_OD) δ 4.40 (m, 1H), 4.22 (m, 1H), 3.58–3.51 (m, 18H), 3.45 (t, 2H, *J* = 5.4), 3.28–3.25 (m, 4H), 3.12 (m, 1H), 2.84 (dd, 1H, *J* = 13.2 and 5.4 Hz), 2.62 (d, 1H, *J* = 13.2 Hz), 2.13 (t, 2H, *J* = 13.2 Hz), 1.67–1.47 (m, 4H), 1.37–1.31 (m, 2H); ^13^C-NMR (150 MHz, CD_3_OD) δ 174.6, 164.6, 70.2, 70.1, 69.8, 69.7, 69.2, 61.9, 60.2, 55.6, 50.4, 39.7, 39.0, 35.3, 28.4, 28.1, 25.5; HRMS (FAB+): mass calculated for C_22_H_41_N_6_O_7_S [M + H]^+^, 533.2679; found, 533.2879. Then, we synthesized *N*-(17-amino-3,6,9,12,15-pentaoxaheptadecyl)-5-((3aS,4S,6aR)-2-oxohexahydro-1H-thieno[3,4-d]imidazol-4-yl)pentanamide (SP-3). A solution of azide (**4**) (29 mg, 0.054 mmol) and 10% Pd/C (6 mg, 0.005 mmol) in MeOH (2 mL) was placed under an atmosphere of hydrogen. After stirring for 24 h, the reaction mixture was diluted with ethyl acetate, filtered through a short pad of celite, and concentrated under reduced pressure. The residue was purified by flash column chromatography on a silica gel (MeOH/CH_2_Cl_2_ = 1:10) to yield amine (**3**) (24 mg, 88%). ^1^H-NMR (600 MHz, CD_3_OD) δ 4.41 (m, 1H), 4.22 (m, 1H), 3.55–3.52 (m, 16H), 3.45–3.41 (m, 4H), 3.27–3.24 (m, 3H), 3.21 (bs, 2H), 3.12 (m, 1H), 2.84 (dd, 1H, *J* = 12.6 and 4.8 Hz), 2.62 (d, 1H, *J* = 12.6 Hz), 2.13 (t, 2H, *J* = 13.2 Hz), 1.66–1.46 (m, 4H), 1.37–1.33 (m, 2H); ^13^C-NMR (150 MHz, CD_3_OD) δ 174.8, 164.6, 72.0, 70.0, 69.9, 69.7, 69.7, 69.4, 61.9, 60.2, 55.6, 40.6, 39.6, 38.8, 35.3, 28.3, 28.1, 25.4; HRMS (FAB+): mass calculated for C_22_H_43_N_4_O_7_S [M + H]^+^, 507.2774; found, 507.2856. Finally, we synthesized *N*1-(4-(*N*-(3,4-dimethylisoxazol-5-yl)sulfamoyl)phenyl)-*N*4-(19-oxo-23-((3aS,4S,6aR)-2-oxohexahydro-1H-thieno[3,4-d]imidazol-4-yl)-3,6,9,12,15-pentaoxa-18-azatricosyl) succinamide (SP-4): To a dimethylformamide (DMF) solution (0.5 mL) of carboxylic acid (**1**) (22 mg, 0.059 mmol), 1-[(1-(cyano-2-ethoxy-2- oxoethylideneaminooxy)-dimethylamino-morpholinomethylene)] methanaminium hexafluorophosphate (COMU) (26 mg, 0.06 mmol) and diisopropylethylamine (DIPEA) (22 μL, 0.129 mmol) were added at 0 °C. After stirring for 30 min, the DMF solution (0.5 mL) of the amine (**3**) (30 mg, 0.059 mmol) was added to the reaction mixture. After stirring for 24 h, the reaction mixture was concentrated under reduced pressure. The residue was purified by flash column chromatography on a silica gel (MeOH/CH_2_Cl_2_ = 1:10) to afford the sulfisoxazole affinity probe (**4**) (25 mg, 49%). ^1^H-NMR (600 MHz, CD_3_OD) δ 7.67 (d, 2H, *J* = 8.4 Hz), 7.63 (d, 2H, *J* = 8.4 Hz), 4.39–4.37 (m, 1H), 4.20 (m, 1H), 3.53–3.50 (m, 16H), 3.43 (d, 4H, *J* = 5.4 Hz), 3.27–3.25 (m, 4H), 3.10 (m, 1H), 2.83 (dd, 1H, *J* = 12.6 and 4.2 Hz), 2.62–2.59 (m, 3H), 2.49 (t, 2H, *J* = 6.6 Hz), 2.12 (t, 2H, *J* = 7.2 Hz), 2.04 (s, 3H), 1.67 (s, 3H), 1.63–1.47 (m, 4H), 1.35–1.33 (m, 2H); ^13^C-NMR (150 MHz, CD_3_OD) δ 174.7, 173.3, 171.9, 164.6, 161.7, 156.9, 143.2, 134.7, 127.8, 118.9, 107.4, 70.1, 70.0, 70.0, 69.8, 69.2, 61.9, 60.2, 55.6, 39.6, 39.0, 38.9, 35.3, 31.6, 30.1, 28.3, 28.1, 25.4, 9.2, 5.0; HRMS (FAB+): mass calculated for C_37_H_58_N_7_O_12_S_2_ [M + H]^+^, 856.3507; found, 856.3580. Chemical structure of sulfisoxazole-based affinity probe is summarized in Supplementary Fig. 13.

### Target identification

To predict the functional targets of SFX, an in silico approach termed Similarity Ensemble Approach (SEA) was used. Of the data registered in BindingDB *(56)*, only those measured through the protein-based assays were extracted. Tanimoto coefficient (Tc) calculated based on Morgan circular fingerprint using the RD Kit (http://www.rdkit.org) was used to quantify the chemical similarity between a test molecule and SFX. The value of Tc was between 0 and 1, indicating that the two molecules with the value closer to 1 shared greater chemical similarity. After retrieval of the protein targets that were small-molecule modulators with the higher Tcs to SFX, SEA was used to compare the similarities. In SEA, the pairwise Tc values between two molecules are summed to form ΣTc. The value of the ΣTc in a test molecule and the distribution of ΣTc in a set of small molecules were drawn with a bar and histogram for comparison.

### Biotin-based pull-down assay

The biotin-based pull-down assay was performed according to the manufacturer’s instructions (#21115; Thermo Scientific). Briefly, a streptavidin ligand-bound agarose gel was immobilized on the biotin-tagged SFX, while the unbound SFX was washed away. Next, membrane proteins of MDA-MB231 cell lysates were incubated with biotin-tagged SFX-bound agarose gel to isolate the prey proteins that were selectively eluted with a low-pH elution buffer and used for western blot analyses.

### Antimicrobial susceptibility testing

The minimal inhibitory concentrations (MICs) of SFX and three structural derivatives were determined by microdilution in Mueller–Hinton agar (#225250; Difco Laboratories) according to the guidelines of the Clinical and Laboratory Standard Institute (CLSI, 2015). *Staphylococcus aureus* (ATCC 29213) and *Escherichia coli* (ATCC 25922) were used as quality control strains.

### Toxicology study and blood chemistry

Seven-week-old male and female ICR mice were supplied by Hanabio Corporation (Seoul, Korea). Animals were bred under SPF conditions and maintained in barrier housing during the experiment. They were maintained in animal care facilities at Chung-Ang University (22 °C ± 1 °C, humidity 60% ± 10%, and a 12 h/12 h light/dark cycle). Nutritionally complete rodent chow and water were provided ad libitum. All protocols were approved by the Ethics Committee of Animal Experiments of Chung-Ang University. This present study was performed according to the general principles of OECD guideline 407 (repeated daily doses for 28-day oral study in rodents) with some modifications. ICR male and female mice were randomly divided into four groups (vehicle control; and low, middle, and high SFX dose groups) according to weight (*n* = 12/subgroup). SFX was suspended in corn oil, and orally administered using an oral gavage needle once a day for 28 days. In a single administration, vehicle control (corn oil), or 100, 300, or 900 mg kg^−1^ day^−1^ of SFX was given to the mice. Mice were weighed every 2 days during the experiment. Individual body weights were used to calculate the correct SFX doses. After collection of blood from the orbital socket, serum from each mouse was stored at −20 °C. Later, serum samples were characterized on a Biochemical Autoanalyzer (Type 7170; Hitachi, Tokyo, Japan) using the following blood chemical parameters: total protein, albumin, globulin, alanine aminotransferase, aspartate aminotransferase, glucose, urea nitrogen, creatinine, low-density lipoprotein, cholesterol, triglyceride, and total bilirubin.

### Cellular assays

For wound healing assay, MDA-MB231 cells were seeded in a 24-well plate and grown to confluence overnight. The next day, the monolayer was wounded by repeated scratches with a 200 μl pipette tip, and the media was changed to remove cell debris. Each wound was imaged at 0 h, and again after 24 h. The average wound healing was assessed by the average of three measurements of the wound areas.

A transwell migration assay was performed using the Costar transwell system (CLS3364; Corning). Briefly, MDA-MB231 cells (2 × 10^3^ cells) were suspended in 200 μl serum-free medium and seeded in the upper insert chamber and 500 μl medium was added to the lower chamber. At 4 h after the cells were seeded, media in both the upper insert and lower chambers were removed. Cells that had migrated into the lower chamber through the 8-μm pore membrane were stained by crystal violet solution and then the migrated cells were visualized using a microscope (×4 magnification).

The cell invasion study was performed using a cell culture insert chamber (CLS3364; Corning). Chamber was coated with the basement membrane Matrigel (100 μl of 20% matrigle/filter, #354248; Corning). The cancer cells were seeded at 10,000 cells/well into upper chamber, and invading cells were fixed and stained with crystal violet. The membranes were mounted on glass slides, and images of the cells were captured using a microscope (×4 magnification).

### 2D colony-forming assay

Cells were seeded in a six-well plate (1 × 10^3^/well) and treated SFX immediately. Then, cells were washed using PBS and treated with the indicated concentrations of SFX every 24 h. After 6 days, cell colonies were fixed with glutaraldehyde (6.0% v/v) and stained with crystal violet (0.5% w/v). Stained colonies were dissolved in 25% methanol, incubated for 10 min, and then measured at 590 nm with a spectrometer (Multiskan^TM^ GO Microplate spectrometer; ThermoFisher).

### Cancer xenograft studies in mice

All animal research was performed in accordance with protocols approved by the Kyungpook National University (KNU) Institutional Animal Care and UCommittes (IACUCs. Approve number: 2017–0146). In the proliferation study, MDA-MB231-luci (+) cells suspended in PBS were orthotopically injected into the left fat pad of 5-week-old female BALB/c *nude* mice. SFX (200 mg kg^−1^ day^−1^) was orally administered for 14 days or zibotentan (10 mg kg^−1^ day^−1^) was intraperitoneally administrated for 14 days. Docetaxel (8 mg kg^−1^ week^−1^) was intravenously administered once a week for two times. Tumor growth was measured every 2 days by using a caliper. After 14 days, mice were euthanized, and luciferase signals were measured using an IVIS imaging system. In rescue experiments, MDA-MB231-luci (+)-derived sEV (10 µg) were intravenously injected once every two days for seven times. In addition, MDA-MB231-ETA K/D cells were orthotopically injected into the left fat pad of 5-week-old female BALB/c nude mice to monitor the cancer progression.

In the metastasis study, 4T1-luci (+) cells suspended in PBS were orthotopically injected into the left fat pad of 5-week-old female BALB/c wild-type mice; other procedures were as described for the proliferation study. Tumor attenuation was monitored every week for 5 weeks using an IVIS imaging system. In each group, five mice were used for the survival test. In addition, Zibotentan (10 mg kg^−1^ day^−1^) or BQ123 (1 mg kg^−1^ day^−1^) were intraperitoneally administrated for 21 days to validate the anti-cancer effect of ETA antagonists. Final attenuation of tumor metastasis was monitored using an IVIS imaging system. In rescue experiments, 4T1-luci (+)-derived sEV (5 μg) were intravenously injected once every 2 days for 11 times.

### Statistical analysis

Unpaired two-tailed students *t*-test was used for experiments comparing two sets of data. The error bars in the graphical data represent means ± standard deviation. All in vitro experiments were performed in triplicates unless otherwise stated. *p* values less than 0.05 were considered to denote statistically significant differences. *, **, and *** denote *p* value of <0.05, 0.005 and 0.001, respectively. NS denotes not significant. Data were analyzed using PRISM 6 software (GraphPad Software, Inc.).

### Reporting Summary

Further information on experimental design is available in the Nature Research Reporting Summary linked to this article.

## Supplementary information

Supplementary Information

Reporting Summary

Source Data

## Data Availability

All microarray data that support the findings of this research have been deposited in the Gene Expression Omnibus (GEO) and are accessible through the GEO accession number GSE117991 (mRNA microarray) and GSE124320 (miRNA microarray). The proteomics data have been deposited to the ProteomeXchange Consortium via the PRIDE with the dataset identifier PXD012689. The source data underlying Figs. 5 (b–g), 2 (b–f), 3 (c, d), 4b–d, i, j), 5 (a, c–g), 6 (b, d, e), 7 (b, d) as well as those underlying Supplementary figs. 1 (b, d–h), 2 (a, b), 3 (c–e, g, h), 5 (a–c), 6 (a), 7 (c–e), 9 (a–d), 10 (a–c), and 11 (b, c, e) are provided as a Source Data file. All other relevant data of this study are available from the corresponding authors upon reasonable request. A reporting summary is available as a Supplementary Information file.
