# Supplementary material for: Sulfisoxazole inhibits the secretion of small extracellular vesicles by targeting the endothelin receptor A
Source: Nat Commun. 2019 Mar 27;10:1387. doi: 10.1038/s41467-019-09387-4 (PMC6437193; doi:10.1038/s41467-019-09387-4)
Supplement: Supplementary file 1 — Supplementary Information [file 41467_2019_9387_MOESM1_ESM.pdf]

Supplementary Information

Sulfisoxazole inhibits the secretion of small extracellular vesicles by targeting the endothelin receptor A

Im et al.

Supplementary Figures

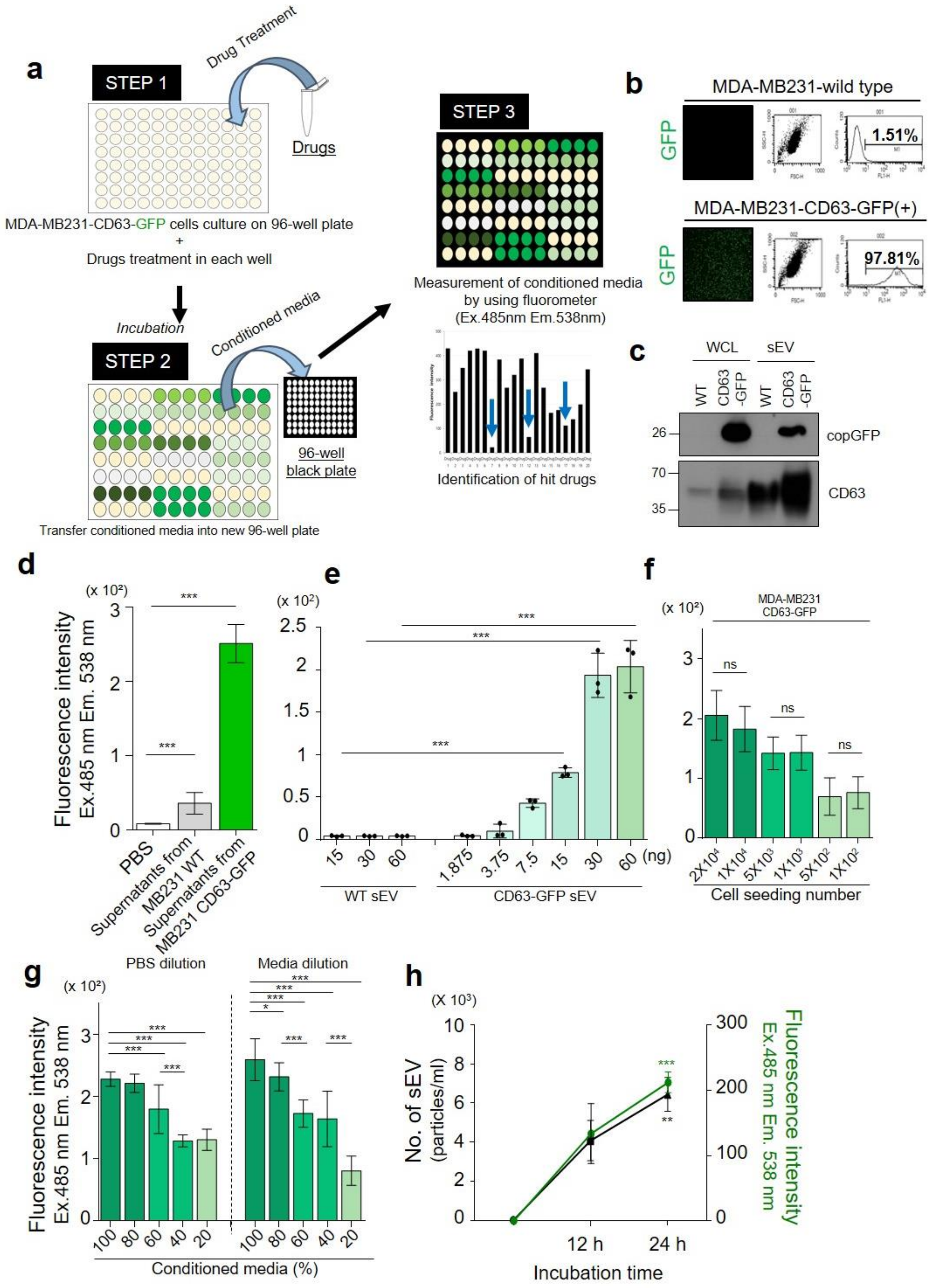

Supplementary Figure 1. High-throughput screening assay to identify an inhibitor of sEV secretion.

(a) Schematic illustration of the high-throughput screening assay system. **(b)** Flow cytometry analysis of WT and CD63-GFP-expressing MDA-MB231 cells (MDA-MB231-CD63-GFP(+) cells. **(c)** Immunoblot of copGFP and CD63 in whole cell lysates and sEV from wild type and MDA-MB231-CD63-GFP(+) cells. **(d)** Measurements of fluorescence intensity in supernatants from wild-type or MDA-MB231-CD63-GFP(+) cells using a fluorometer. (Ex. 480 nm; Em. 538 nm) (*n*=15). **(e)** Measurement of fluorescence intensity from WT and CD63-GFP. Different amounts of sEV (15 to 60 ng) were added into the medium (phenol-negative DMEM). **(f)** Measurement of fluorescence intensity in the supernatant from MDA-MB231-CD63-GFP(+) cells (*n*=24). **(g)** Measurement of fluorescence intensity in the supernatant from MDA-MB231-CD63-GFP(+) cells. Supernatants were diluted in different volumes of PBS or medium to indicate relative percentage (*n*=16). **(h)** Comparison of the results derived from fluorescence with those from NTA in MDA-MB231-CD63-GFP (+) cells. MDA-MB231-CD63-GFP (+) cells were cultured for indicated times. The sEV and supernatants were prepared for counting sEV and calculating fluorescence, respectively, as described in the Method Section. Green, GFP intensity. Black, the number of sEV. Experiments were performed with 95% confluent cells**.** Significance was determined using an unpaired two-tailed Student's t test. ***$p<0.001$, **$p<0.005$ and *$p<0.05$. Error Bar, S.D. Source data (b, d-h) are provided as a Source Data file.

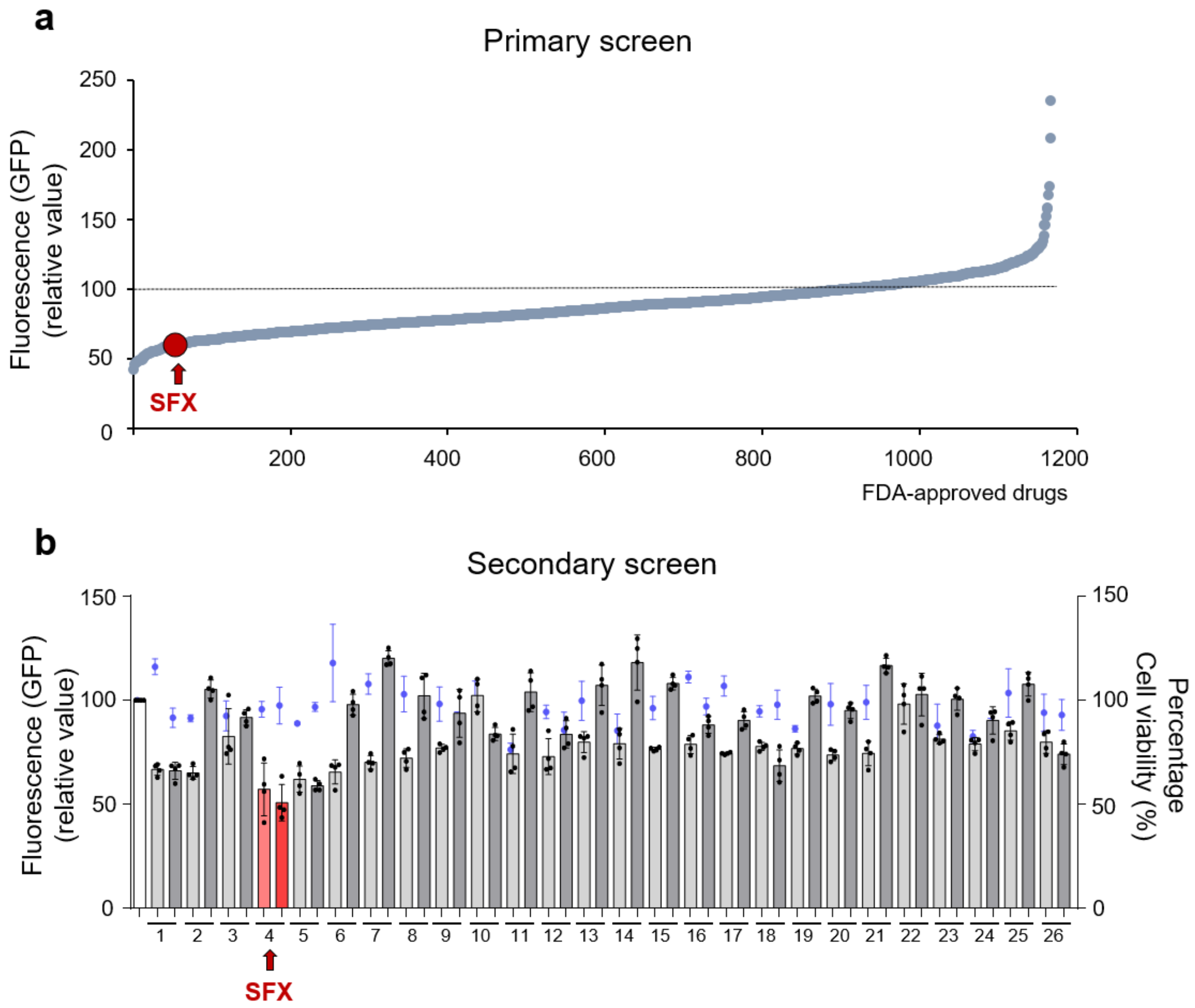
a
Primary screen
Fluorescence (GFP) (relative value)
250
200
150
100
50
0
SFX
200
400
600
800
1000
1200
FDA-approved drugs
b
Secondary screen
Fluorescence (GFP) (relative value)
150
100
50
0
Percentage Cell viability (%)
150
100
50
0
1
2
3
4
5
6
7
8
9
10
11
12
13
14
15
16
17
18
19
20
21
22
23
24
25
26
SFX

Supplementary Figure 2. High-throughput screening assay to identify a sEV inhibitor, SFX.

(a) Measurement of GFP fluorescence intensity in the supernatants from MDA-MB231-CD63-GFP (+) cells treated with 1,163 FDA-approved drugs. Drug concentrations were 30 μM for the primary screening. **(b)** For the secondary screening, MDA-MB231 CD63-GFP (+) cells were treated with 50 or 100 μM of 26 drugs. Blue dot, percentage cell viability. Bar, Fluorescence (GFP) intensity (relative value). (*n*=3) The secondary screening was performed in triplicates. Experiments were performed with 95% confluent cells. Source data (a-b) are provided as a Source Data file.

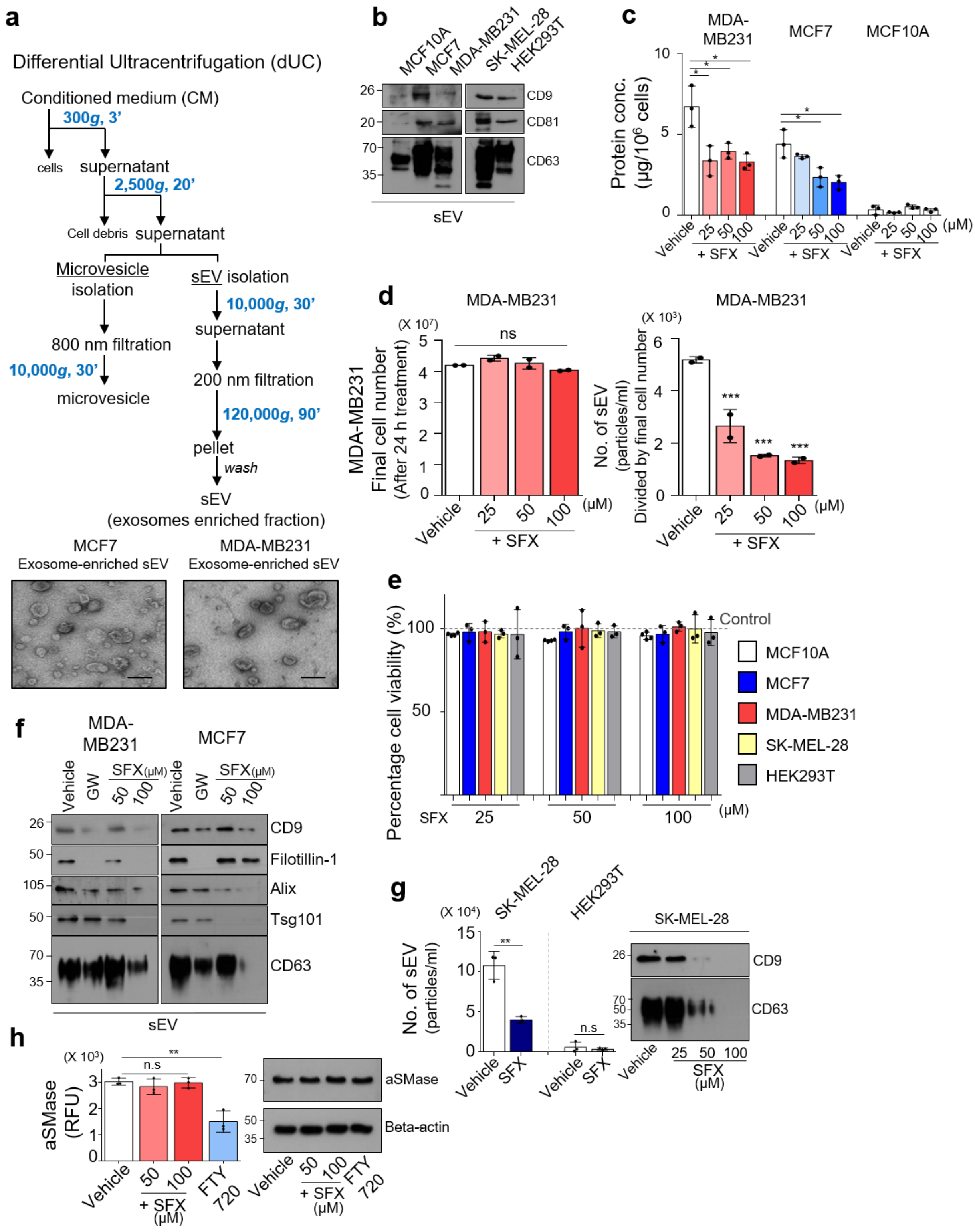
a
Differential Ultracentrifugation (dUC)
Conditioned medium (CM)
300g, 3’
cells
supernatant
2,500g, 20’
Cell debris
supernatant
Microvesicle isolation
sEV isolation
10,000g, 30’
supernatant
800 nm filtration
10,000g, 30’
microvesicle
200 nm filtration
120,000g, 90’
pellet
wash
sEV
(exosomes enriched fraction)
MCF7
Exosome-enriched sEV
MDA-MB231
Exosome-enriched sEV
b
MCF10A
MCF7
MDA-MB231
SK-MEL-28
HEK293T
CD9
CD81
CD63
sEV
c
MDA-MB231
MCF7
MCF10A
Protein conc. (μg/10^6 cells)
Vehicle
25
50
100
(μM)
+ SFX
d
MDA-MB231
ns
(X 10^7)
MDA-MB231
Final cell number
(After 24 h treatment)
(X 10^3)
No. of sEV
(particles/ml)
Divided by final cell number
***
e
Percentage cell viability (%)
Control
MCF10A
MCF7
MDA-MB231
SK-MEL-28
HEK293T
SFX
(μM)
f
MDA-MB231
MCF7
Vehicle
GW
SFX(μM)
CD9
Filotillin-1
Alix
Tsg101
CD63
sEV
g
SK-MEL-28
HEK293T
(X 10^4)
No. of sEV
(particles/ml)
**
n.s
Vehicle
SFX
CD9
CD63
SFX
(μM)
h
(X 10^3)
aSMase
(RFU)
n.s
**
Vehicle
+ SFX
(μM)
FTY 720
aSMase
Beta-actin

Supplementary Figure 3. SFX-mediated blockade of the sEV biogenesis and secretion pathway.

(a) Illustration of the differential ultracentrifugation methods used to isolate microvesicles and small extracellular vesicles (sEV). Bottom, Images of exosome-enriched sEV isolated from MCF7 or MDA-MB231 cells analyzed by TEM. Scale bar, 100 nm. **(b)** Immunoblot of CD9, CD81 and CD63 proteins in sEV from MCF10A, MCF7, MDA-MB231, SK-MEL-28, and HEK293T. **(c)** Measurements of the sEV protein from MDA-MB231, MCF7, and MCF10A cells. (*n*=3) **(d)** Measurement of final cell number after SFX treatment (left) and the calculated number of sEV (right) *(n=2).* **(e)** Measurement of cell viability of the indicated normal and cancer cells. Normal cell lines; MCF10A and HEK293T. Cancer cell lines; MDA-MB231, MCF7 and SK-MEL-28. Broken line, control value. (*n*=3) **(f)** Immunoblot of various sEV proteins. sEV were prepared from the equal numbers of cells. GW4869 was used as a positive control. (**g)** Left, The number of sEV secreted from SK-MEL-28 and HEK293T cells for 24 h in the presence of 100 μM SFX. Right, Immunoblot of CD9 and CD63 proteins in sEV secreted from SK-MEL-28 after treatment with the different concentrations of SFX. (*n*=3) **(h)** Immunoblot (Right) and activity (Left) of acidic sphingomyelinase (aSMase) in MDA-MB231 cells. FTY720, a known aSMase inhibitor, was used as positive control. (*n*=3) Experiments were performed with 95% confluent cells. Significance was determined using an unpaired two-tailed Student's t test. **$p<0.005$. n.s, Not significant. Error Bar, S.D. Source data (c-e, g-h) are provided as a Source Data file.

a

Down regulated sEV miRNA
MDA-MB231
(SFX vs Vehicle)

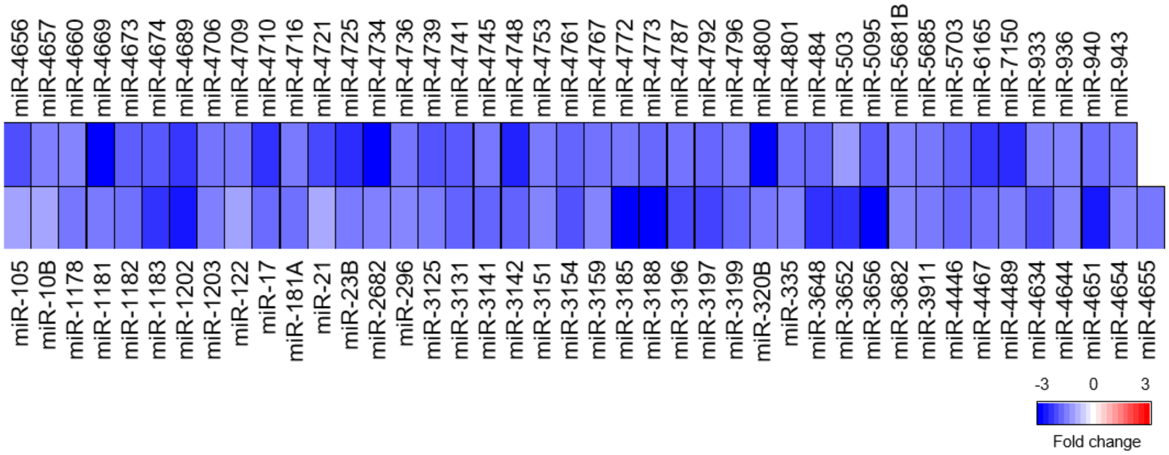

b

Down-regulated sEV protein
MDA-MB231
(SFX vs Vehicle)

Accession	Gene name	Protein name	SFX/Control Ratio
O00560	SDCB1	Syntenin-1	0.1
Q13733	AT1A4	Sodium/potassium-transporting ATPase subunit alpha-4	0.1
Q02241	KIF23	Kinesin-like protein KIF23	0.1
P55268	LAMB2	Laminin subunit beta-2	0.11
O43854	EDIL3	EGF-like repeat and discoidin I-like domain-containing protein 3	0.12
P04899	GNAI2	Guanine nucleotide-binding protein G(i) subunit alpha-2	0.12
P39060	COIA1	Collagen alpha-1(XVIII) chain	0.12
P0DMV8	HS71A	Heat shock 70 kDa protein 1A	0.12
P0DMV9	HS71B	Heat shock 70 kDa protein 1B	0.12
P98088	MUC5A	Mucin-5AC	0.13
P29317	EPHA2	Ephrin type-A receptor 2	0.13
P98160	PGBM	Basement membrane-specific heparan sulfate proteoglycan core protein	0.13
P60033	CD81	CD81 antigen	0.13
Q15286	RAB35	Ras-related protein Rab-35	0.13
P26006	ITA3	Integrin alpha-3	0.14
P23229	ITA6	Integrin alpha-6	0.17
P16070	CD44	CD44 antigen	0.17
P07355	ANXA2	Annexin A2	0.19
P14618	KPYM	Pyruvate kinase PKM	0.19
P08238	HS90B	Heat shock protein HSP 90-beta	0.2
P00533	EGFR	Epidermal growth factor receptor	0.2
Q99715	COCA1	Collagen alpha-1(XII) chain	0.23
Q08431	MFGM	Lactadherin	0.23
Q08380	LG3BP	Galectin-3-binding protein	0.25
P35527	K1C9	Keratin type I cytoskeletal 9	0.34
P13645	K1C10	Keratin type I cytoskeletal 10	0.37
P13473	LAMP2	Lysosome-associated membrane glycoprotein 2	0.38
P07996	TSP1	Thrombospondin-1	0.52

Supplementary Figure 4. SFX-mediated miRNA and protein cargo reduction.

(a) A heatmap of the selected sEV miRNA of SFX-treated MDA-MB231 cells, compared to untreated control cells. The heatmap represents the probe sets for miRNAs expressed at significantly lower levels after treatment with 100 μM SFX, respectively. The GEO accession number of NCBI is GSE124320. **(b)** Selected sEV proteins of SFX-treated MDA-MB231 cells, compared to untreated control cells (false discovery rate, FDR < 5%). SFX/Control ratio was calculated and summarized. The accession number of ProteomeXchange Consortium is PXD012689.

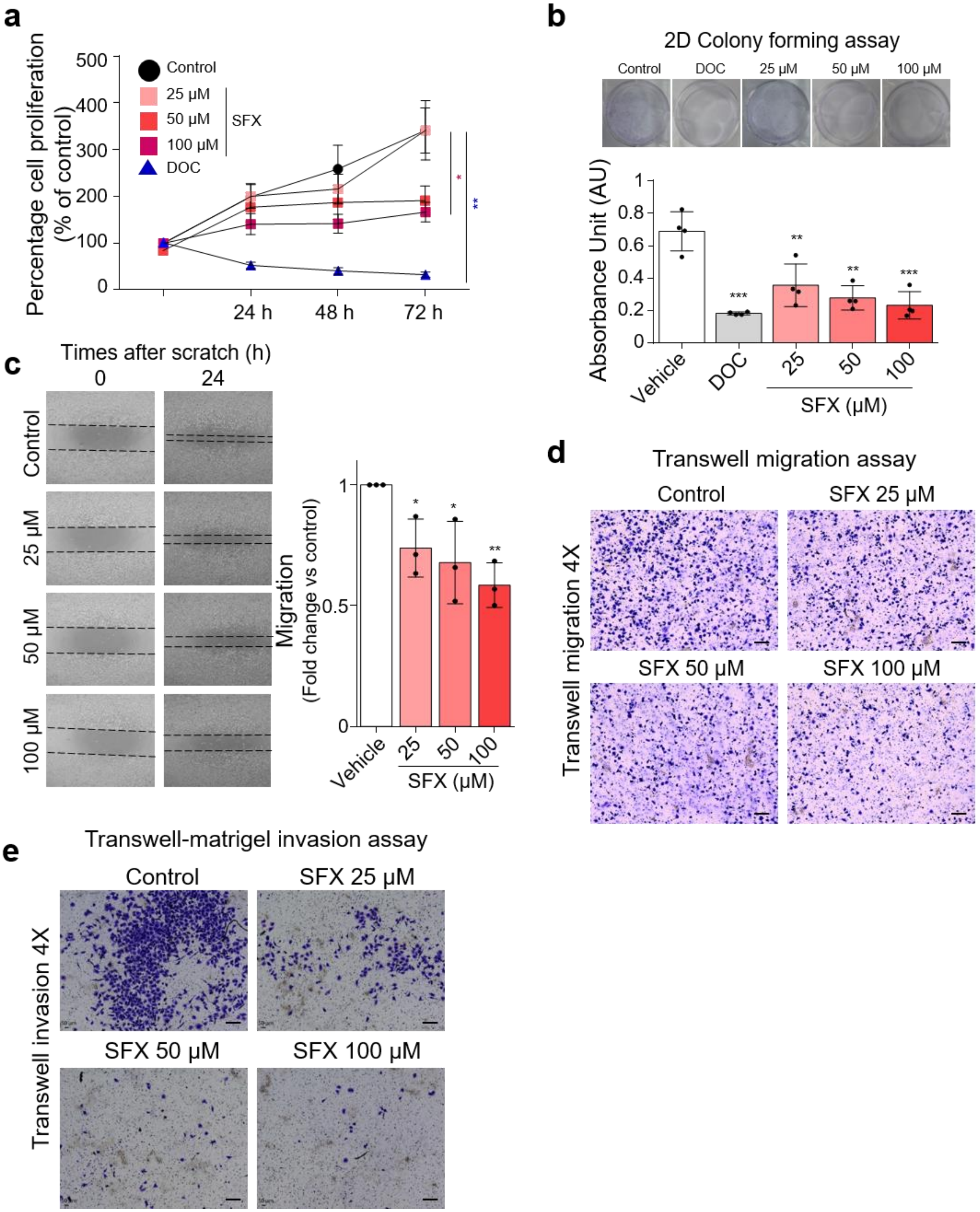

a
Percentage cell proliferation (% of control)
500
400
300
200
100
0
Control
25 µM
50 µM
100 µM
SFX
DOC
24 h
48 h
72 h
*
**
b
2D Colony forming assay
Control
DOC
25 µM
50 µM
100 µM
Absorbance Unit (AU)
1
0.8
0.6
0.4
0.2
0
***
**
**
***
Vehicle
DOC
25
50
100
SFX (µM)
c
Times after scratch (h)
0
24
Control
25 µM
50 µM
100 µM
Migration (Fold change vs control)
1
0.5
0
*
*
**
Vehicle
25
50
100
SFX (µM)
d
Transwell migration assay
Control
SFX 25 µM
SFX 50 µM
SFX 100 µM
Transwell migration 4X
e
Transwell-matrigel invasion assay
Control
SFX 25 µM
SFX 50 µM
SFX 100 µM
Transwell invasion 4X

Supplementary Figure 5. SFX-mediated suppression of cancer cell activities.

(a) Measurement of cancer cell proliferation. MDA-MB231 cells were treated with the indicated concentrations of SFX or 10 nM docetaxel (DOC) every 24 h for up to 3 days. After 24, 48 or 72 h, rates of cell proliferation were measured by MTT reduction method. DOC was used as a positive control. The population of starting cells for this experiment should be a low confluency of about 8%. **(b)** Analysis of the 2D colonic forming assay for SFX-treated MDA-MB231 cells. The population of starting cells was a low confluency of about 8%. **(c)** Analysis of a wound-healing assay in SFX-treated MDA-MB231 cells. Broken line, wound area. **(d)** Analysis of the transwell migration assay in SFX-treated MDA-MB231 cells. Migrated cells were stained using crystal violet solution and random fields were captured. Magnification, ×4. Scale bar, 50 μm. **(e)** Analysis of a matrigel-coated transwell invasion assay in SFX-treated MDA-MB231 cells. Magnification, ×4. Scale bar, 50 μm. All experiments were performed in triplicates. Experiments were performed with 95% confluent cells except for the proliferation and colony forming assays. Significance was determined using an unpaired two-tailed Student's t test. ***$p<0.001$, **$p<0.005$ and *$p<0.05$. Error Bar, S.D. Source data (a-c) are provided as a Source Data file.

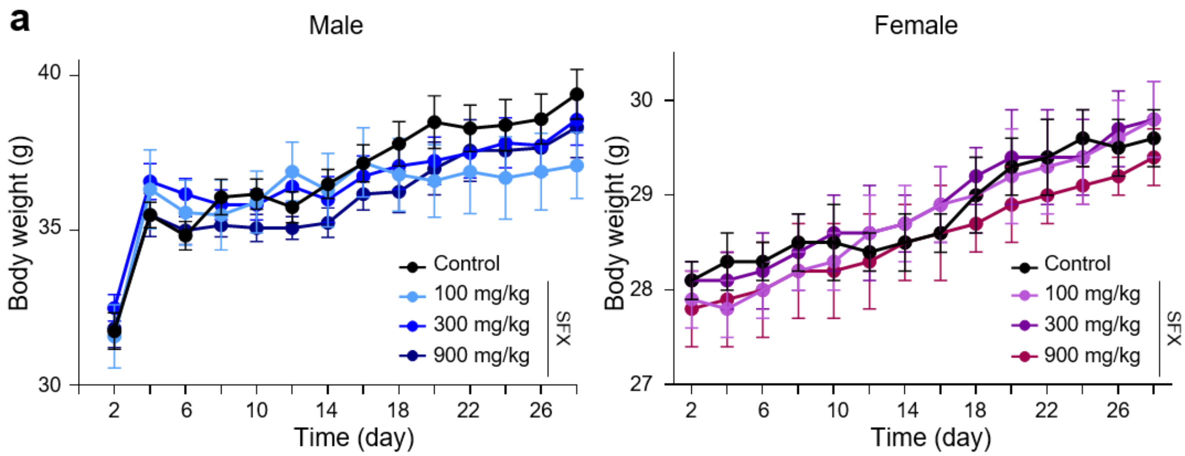

b Serum chemistry following a 28-repeated dose toxicity study of SFX in mice

parameter	male (mg/kg)				female (mg/kg)			
	control	100	300	900	control	100	300	900
AST	64.3	62.4	53.0	57.0	74.5	71.3	72.2	67.5
(IU/L)	(10.4)	(9.5)	(10.1)	(16.9)	(21.4)	(28.7)	(23.6)	(15.5)
ALT	39.1	46.3	32.3	28.9	45.1	34.4	37.9	27.1
(IU/L)	(18.4)	(19.9)	(17.3)	(9.2)	(11.6)	(16.7)	(28.2)	(5.7)
Glucose	242.3	277.2	282.5	278.5	268.2	241.2	246.8	222.8
(mg/dl)	(30.0)	(51.1)	(50.2)	(31.9)	(60.7)	(18.7)	(27.0)	(19.7)
Tot_P	4.63	4.75	4.70	4.47	4.80	4.80	4.75	4.83
(g/dl)	(0.31)	(0.41)	(0.32)	(0.21)	(0.19)	(0.38)	(0.27)	(0.12)
Albumin	1.50	1.52	1.52	0.45	1.65	1.58	1.63	1.65
(g/dl)	(0.11	(0.13)	(0.12)	(0.10	(0.08)	(0.08)	(0.08)	(0.05)
Globulin	3.13	3.23	3.18	3.02	3.15	3.22	3.12	3.18
(g/dl)	(0.23)	(0.33)	(0.21)	(0.12)	(0.14)	(0.40)	(0.20)	(0.10)
TG	58.2	38.3	44.5	42.5	35.8	27.5	26.0	34.5
(mg/dl)	(21.1)	(15.6)	(14.9)	(18.6)	(21.2)	(12.1)	(16.4)	(7.6)
LDL	5.83	6.00	7.83	8.00	6.17	8.83	7.17	7.33
(mg/dl)	(1.94)	(1.26)	(2.14)	(3.52)	(1.47)	(2.48)	(1.33)	(1.37)
BUN	25.2	20.4	20.3	20.0	20.9	18.5	17.4	17.4
(mg/dl)	(4.0)	(1.7)	(1.6)	(1.8)	(2.9)	(2.8)	(1.5)	(3.6)
Scr	0.38	0.38	0.36	0.34	0.39	0.38	0.36	0.33
(mg/dl)	(0.03)	(0.03)	(0.03)	(0.02)	(0.01)	(0.03)	(0.02)	(0.02)
Tot_B	0.14	0.13	0.13	0.15	0.07	0.09	0.10	0.09
(mg/dl)	(0.06)	(0.04)	(0.02)	(0.04)	(0.01)	(0.02)	(0.02)	(0.02)

AST: aspartate transaminase, ALT: alanine transaminase, Tot_P: total protein, TG: triglyceride, LDL: low-density lipoprotein, BUN: blood urea nitrogen, Scr: serum creatinine, Tot_B: total bilirubin; data are mean (s.d.) (n=12).

Supplementary Figure 6. No apparent sub-acute toxicities by SFX

(a) Body weight changes in male and female mice exposed to the different concentrations of SFX for 28 days (*n*=12/group). Body weight for each mouse was measured every 2 days. **(b)** Serum chemistry following a 28-repeated dose toxicity study of SFX in mice (*n*=12/group). Brace, S.D. of each parameter. Source data are provided as a Source Data file. Source data (a) is provided as a Source Data file.

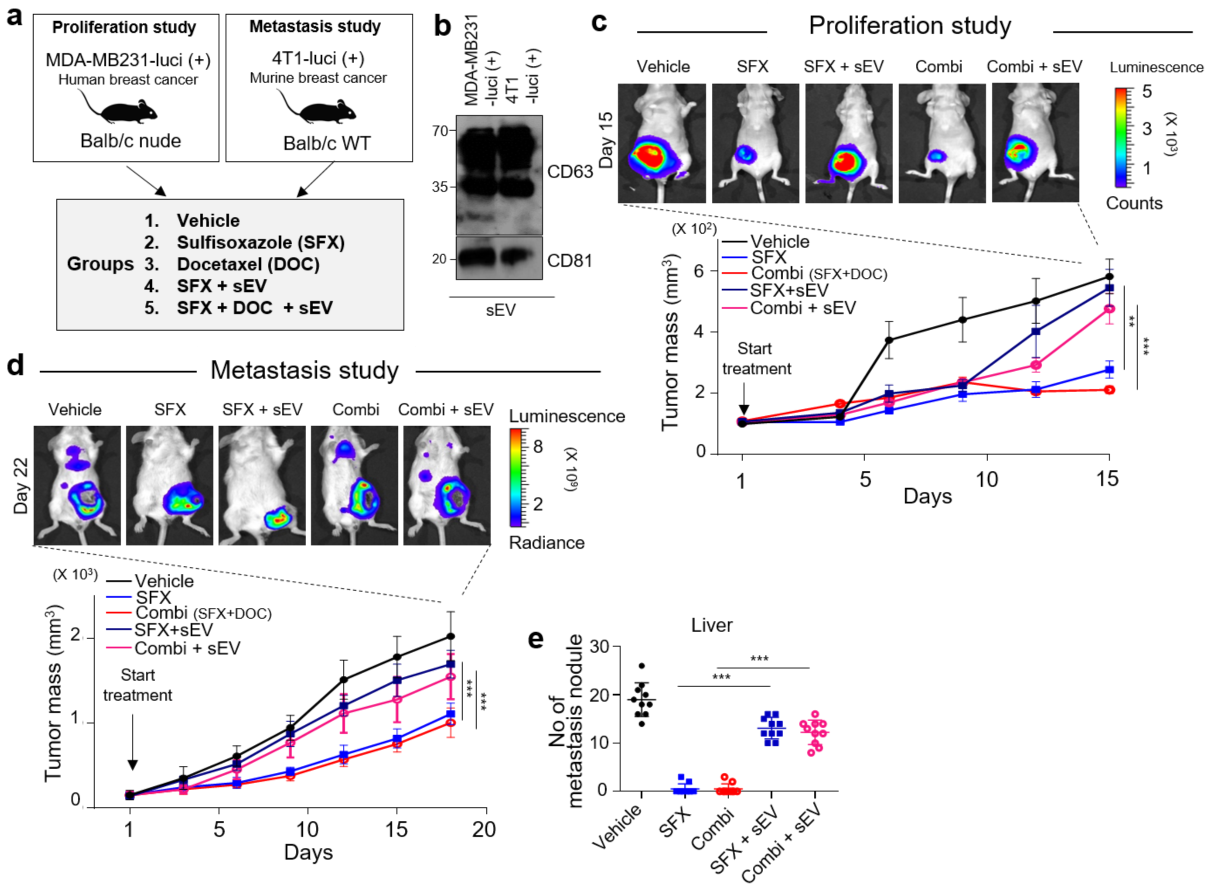
a
Proliferation study
MDA-MB231-luci (+)
Human breast cancer
Balb/c nude
Metastasis study
4T1-luci (+)
Murine breast cancer
Balb/c WT
Groups
1. Vehicle
2. Sulfisoxazole (SFX)
3. Docetaxel (DOC)
4. SFX + sEV
5. SFX + DOC + sEV
b
MDA-MB231 -luci (+)
4T1 -luci (+)
70
35
20
CD63
CD81
sEV
c
Proliferation study
Vehicle
SFX
SFX + sEV
Combi
Combi + sEV
Day 15
Luminescence
5
3
1
(X 10³)
Counts
(X 10²)
Vehicle
SFX
Combi (SFX+DOC)
SFX+sEV
Combi + sEV
Tumor mass (mm³)
Start treatment
Days
d
Metastasis study
Vehicle
SFX
SFX + sEV
Combi
Combi + sEV
Day 22
Luminescence
8
2
(X 10⁹)
Radiance
(X 10³)
Vehicle
SFX
Combi (SFX+DOC)
SFX+sEV
Combi + sEV
Tumor mass (mm³)
Start treatment
Days
e
Liver
No of metastasis nodule
Vehicle
SFX
Combi
SFX + sEV
Combi + sEV

Supplementary Figure 7. SFX suppresses breast cancer cell proliferation and metastasis via sEV modulation.

(a) Schematic illustration of the *in vivo* experimental designs with different treatments. **(b)** Immunoblot of CD63 and CD81 in sEV from MDA-MB231-luci (+) and 4T1-luci (+) cells. (c). Top, Representative image of cancer cells tracked with the IVIS imaging system following the injection of mice with luciferase-expressing MDA-MB231 cells. Bottom, Tumor mass volume of in BALB/c *nude* female mice inoculated with MDA-MB231-luci (+) cells and then treated with an indicated drug, vehicle and sEV (n=8 per group). **(d)** Top, *in vivo* images of 4T1 cancer cell-bearing mice using an IVIS imaging system. Bottom, Tumor mass volume of 4T1-luci (+) cells inoculated BALB/c *nude* female mice treated with an indicated drug, vehicle and sEV (n=10 per group). **(d)** Quantitative representation of liver nodules from mice bearing 4T1-luci (+) cells (n=10 per group). Significance was determined using an unpaired two-tailed Student's t test. ***p<0.001, **p<0.005 and *p<0.05. Error Bar, S.D. Source data (c-e) are provided as a Source Data file.

a

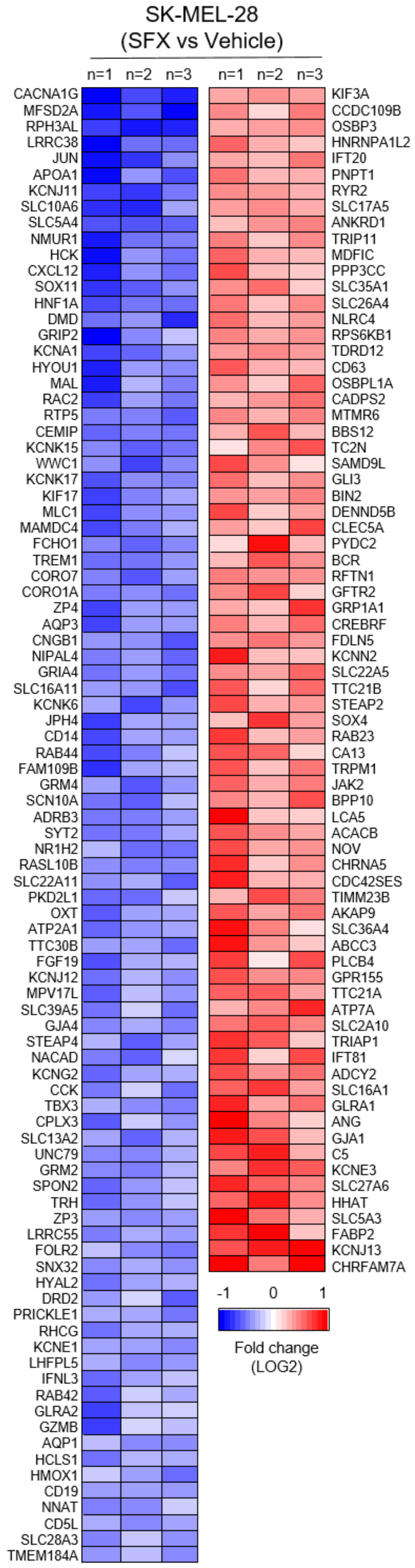

b

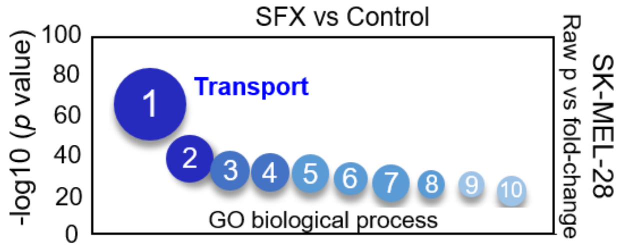

c

RANK	MDA-MB231	SK-MEL-28
	TERM	TERM
1	Transport	Transport
2	Small GTPase mediated signal transduction	Regulation of transport
3	Regulation of transport	Positive regulation of transport
4	Vesicle-mediated transport	Secretion
5	Secretion	Secretion by cell
6	Secretion by cell	Regulation of secretion
7	Endomembrane system organization	Vesicle-mediated transport
8	Endocytosis	Positive regulation of secretion
9	Positive regulation of transport	Positive regulation of secretion by cell
10	Regulation of secretion by cell	Endocytosis

Supplementary Figure 8. SFX-mediated selective alteration of the genes involved in sEV secretion in SK-MEL-28 melanoma cells.

(a) A heatmap of the selected transcriptome of SFX-treated SK-MEL-28 cells, compared to untreated control cells. The heatmap represents the probe sets for transcripts expressed at significantly higher or lower levels after treatment with 100 μM SFX, respectively (FC = ± 1.3 *p*-value = 0.05). **(b)** The advanced bubble chart shows the enrichment of differentially expressed genes in the indicated signaling pathways. The *x*-axis label indicates the rank of GO biological processes, and the *y*-axis label represents *p* value. The sizes of the bubble represent the amounts of differentially expressed genes enriched in biological processes. **(c)** The rank of GO biological processes was summarized. Microarray was performed in triplicates. The GEO accession number of NCBI dataset is GSE117991.

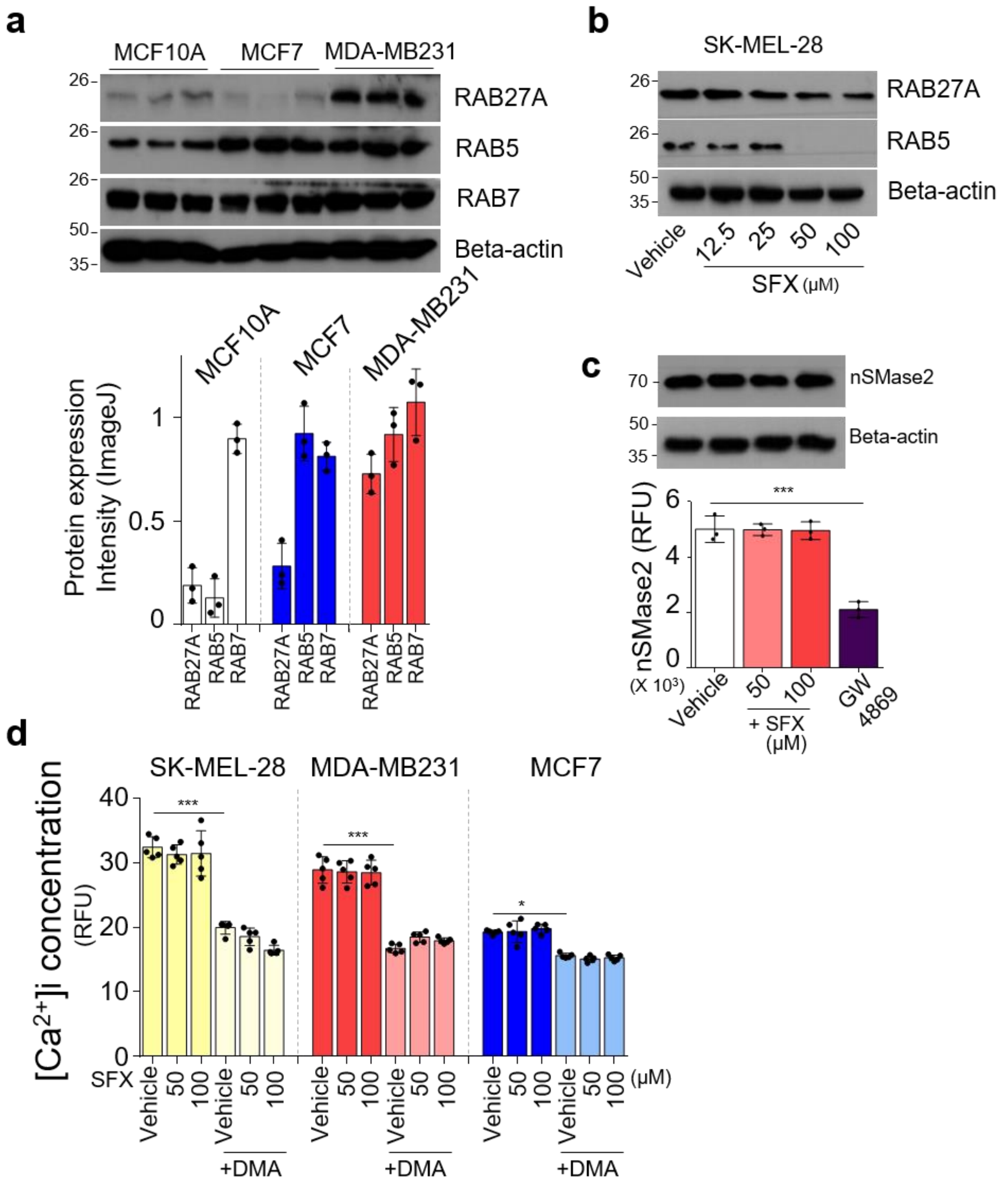

a
MCF10A
MCF7
MDA-MB231
RAB27A
RAB5
RAB7
Beta-actin
Protein expression Intensity (ImageJ)
b
SK-MEL-28
RAB27A
RAB5
Beta-actin
Vehicle
12.5
25
50
100
SFX (μM)
c
nSMase2
Beta-actin
nSMase2 (RFU)
(X 10³)
Vehicle
+ SFX (μM)
GW 4869
d
SK-MEL-28
MDA-MB231
MCF7
[Ca²⁺]i concentration (RFU)
SFX
Vehicle
50
100
(μM)
+DMA

Supplementary Figure 9. SFX-mediated blockade of the sEV biogenesis/secretion pathway through the ESCRT-dependent pathway.

(a) Differential expression of the sEV biogenesis-related proteins in three breast cells. Basal expression levels of Rab27a, Rab5, and Rab7 in MCF10A, MCF7, and MDA-MB231 cells were measured by western blot analysis (n=3 per group). **(b)** Immunoblot of RAB27A and RAB5 in SK-MEL-28 melanoma cells treated with the indicated concentrations of SFX. **(c)** Immunoblot (top) and activity (bottom) of neutral sphingomyelinase-2 (nSMase2) in MDA-MB231 cells. GW4869, an inhibitor of nSMase2, was used as a positive control. **(d)** Measurement of intracellular calcium concentrations by Fluo-3AM method in SK-MEL-28, MCF7 and MDA-MB231 cells. Dimethyl amiloride (DMA) was used as a positive control. (n=5). All experiments were performed in triplicates. Experiment were performed using 95 % confluency of cells. Significance was determined using an unpaired two-tailed Student's t test. ***p<0.001 and *p<0.05. Error Bar, S.D. Source data (a-d) are provided as a Source Data file.

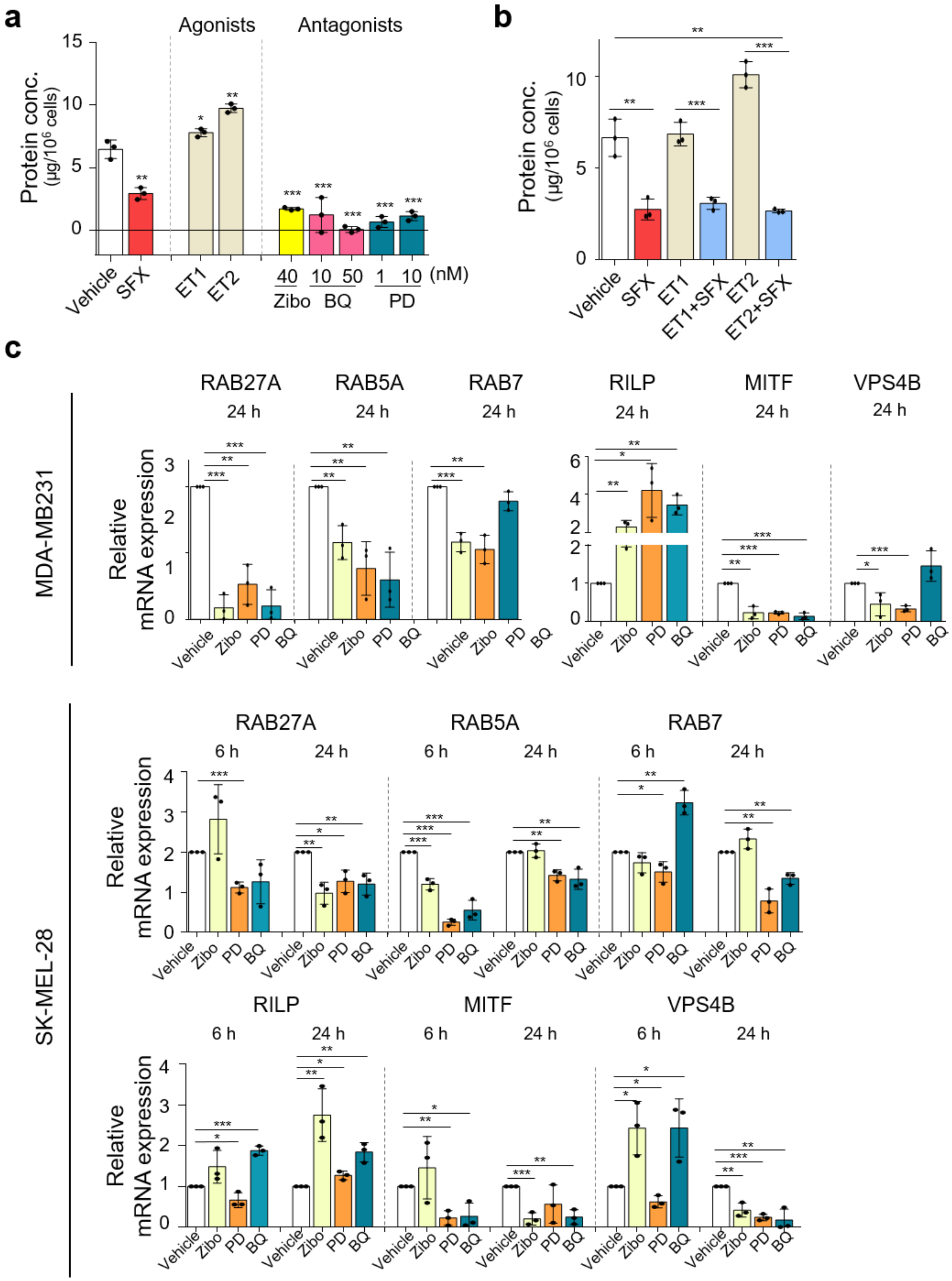

a
Agonists
Antagonists
Protein conc.
(μg/10^6 cells)
Vehicle
SFX
ET1
ET2
40
Zibo
10 50
BQ
1 10
PD
(nM)
b
Protein conc.
(μg/10^6 cells)
Vehicle
SFX
ET1
ET1+SFX
ET2
ET2+SFX
c
MDA-MB231
RAB27A
RAB5A
RAB7
RILP
MITF
VPS4B
24 h
Relative
mRNA expression
Vehicle
Zibo
PD
BQ
SK-MEL-28
6 h
24 h

Supplementary Figure 10. ETA antagonists-mediated blockade of the mRNA expression levels of the proteins associated with sEV biogenesis and secretion.

(a) Measurement of the amount of sEV proteins from ETA agonist- or antagonist-treated MDA-MB231 cells. **(b)** Measurement of the amounts of sEV proteins from MDA-MB231 cells treated with 10 nM ET1 or 10 nM ET2 in the presence of 100 μM SFX. **(c)** Changes in the expression of RAB27A, RAB5A, RAB7, RILP, MITF, and VPS4B mRNA transcripts in MDA-MB231 and SK-MEL-28 cells by the indicated ETA antagonists. The levels of expressed genes were calculated by the delta-delta Ct (△△Ct) method. Zibo, 40 nM Zibotentan. PD, 10 nM PD156707. BQ, 50 nM BQ123. All experiments were performed in triplicates. Experiment were performed using 95 % confluency of cells. Significance was determined using an unpaired two-tailed Student's t test. ****p*<0.001, ***p*<0.005 and **p*<0.05. Error Bar, S.D. Source data (a-c) are provided as a Source Data file.

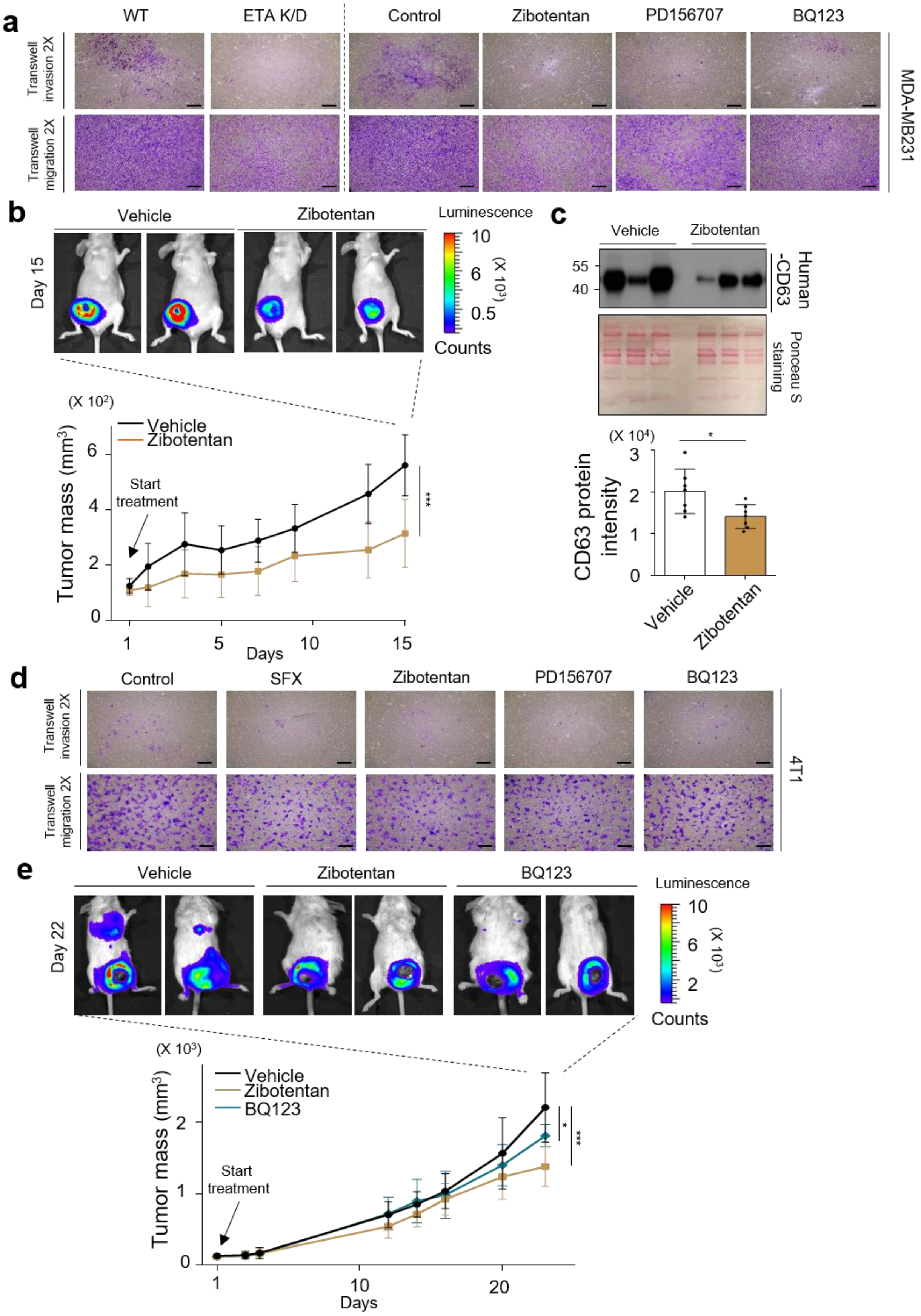

a
WT
ETA K/D
Control
Zibotentan
PD156707
BQ123
Transwell invasion 2X
Transwell migration 2X
MDA-MB231
b
Vehicle
Zibotentan
Day 15
Luminescence
10
6
0.5
(X 10³)
Counts
(X 10²)
Vehicle
Zibotentan
Tumor mass (mm³)
Start treatment
Days
***
c
Vehicle
Zibotentan
55
40
Human -CD63
Ponceau S staining
(X 10⁴)
CD63 protein intensity
*
d
Control
SFX
Zibotentan
PD156707
BQ123
Transwell invasion 2X
Transwell migration 2X
4T1
e
Vehicle
Zibotentan
BQ123
Day 22
Luminescence
(X 10³)
Counts
Tumor mass (mm³)
Start treatment
Days
*
***

Supplementary Figure 11. ETA mediated suppression of cancer cell activities

(a) Analysis of the transwell invasion (upper) and migration (bottom) assays in ETA antagonists-treated MDA-MB231 cells and MDA-MB231 ETA K/D cells. Invaded or migrated cells were stained using crystal violet solution and random fields were captured. Magnification, ×2. Scale bar, 100 μm. **(b)** Top, Representative image of cancer cells tracked with the IVIS imaging system following the injection of mice with luciferase-expressing MDA-MB231 cells. Bottom, Tumor mass volume of in BALB/c *nude* female mice inoculated with MDA-MB231-luci (+) cells and then treated with an indicated drug or vehicle (n=9 per group). **(c)** Upper, Immunoblot of human CD63 in circulating cancer-cell derived sEV from MDA-MB231-luci (+)-bearing mice (n=6 per group) where similar amounts of protein/lane were verified by Ponceau S staining (Bottom). Bottom, Analysis of the relative protein intensity of CD63 measured using a densitometer system. **(d)** Analysis of the transwell invasion (upper) and migration (bottom) assays in ETA antagonists-treated 4T1 cells. Invaded or migrated cells were stained using crystal violet solution and random fields were captured. Magnification, ×2. Scale bar, 100 μm. **(e)** Top, *in vivo* images of 4T1 cancer cell-bearing mice using an IVIS imaging system. Bottom, Tumor mass volume of 4T1-luci (+) cells inoculated BALB/c *nude* female mice treated with an indicated drug or vehicle (n=9 per group). Experiment were performed using 95 % confluency of cells. Significance was determined using an unpaired two-tailed Student's t test. ***p<0.001, **p<0.005 and *p<0.05. Error Bar, S.D. Source data are provided as a Source Data file. Source data (b-c, e) are provided as a Source Data file.

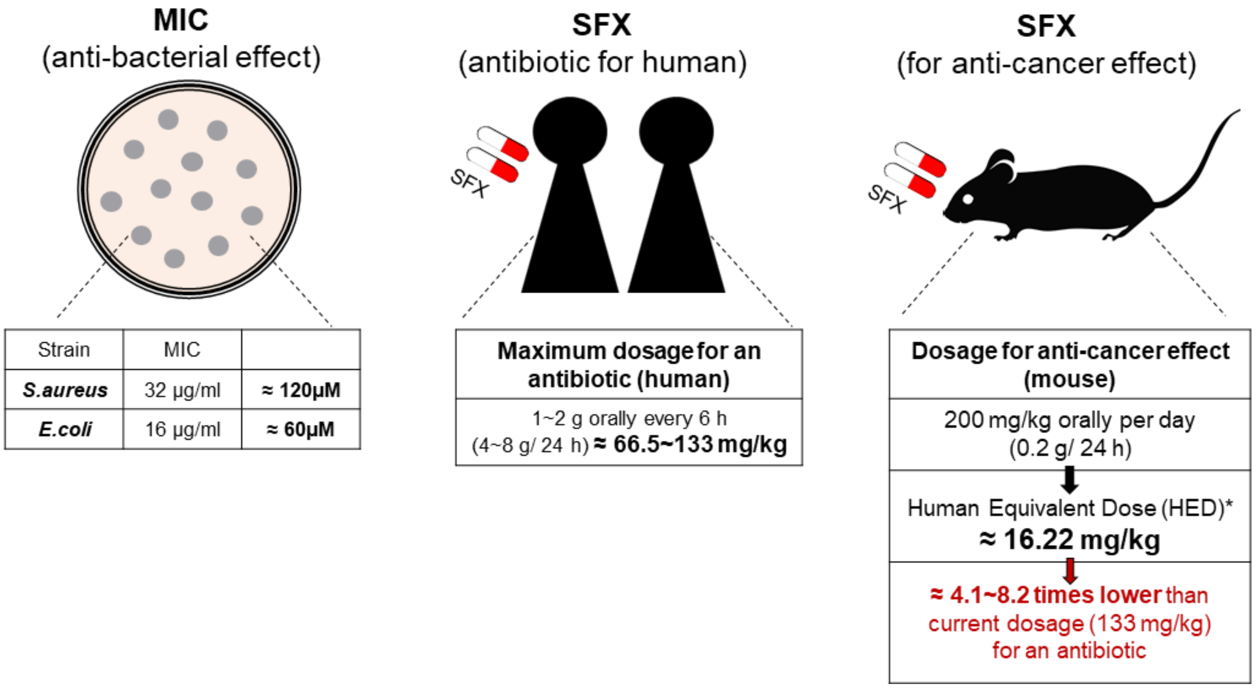
MIC
(anti-bacterial effect)
Strain
MIC
S.aureus
32 μg/ml
≈ 120μM
E.coli
16 μg/ml
≈ 60μM
SFX
(antibiotic for human)
SFX
Maximum dosage for an antibiotic (human)
1~2 g orally every 6 h
(4~8 g/ 24 h) ≈ 66.5~133 mg/kg
SFX
(for anti-cancer effect)
SFX
Dosage for anti-cancer effect (mouse)
200 mg/kg orally per day
(0.2 g/ 24 h)
Human Equivalent Dose (HED)*
≈ 16.22 mg/kg
≈ 4.1~8.2 times lower than
current dosage (133 mg/kg)
for an antibiotic

Supplementary Figure 12. Clinical relevance of SFX dosages.

The unit (μg ml^{-1}) of MIC converted to the μM unit. The recommended dosage for an antibiotic in human usage is about 66.5 to 133 mg kg^{-1}. The dosage (200 mg kg^{-1}) for mouse study is converted to a human equivalent dose (approximately 16.22 mg kg^{-1}).

Supplementary Figure 13. Chemical structure of sulfisoxazole-based affinity probe

Synthesis of a sulfisoxazole-based affinity probe

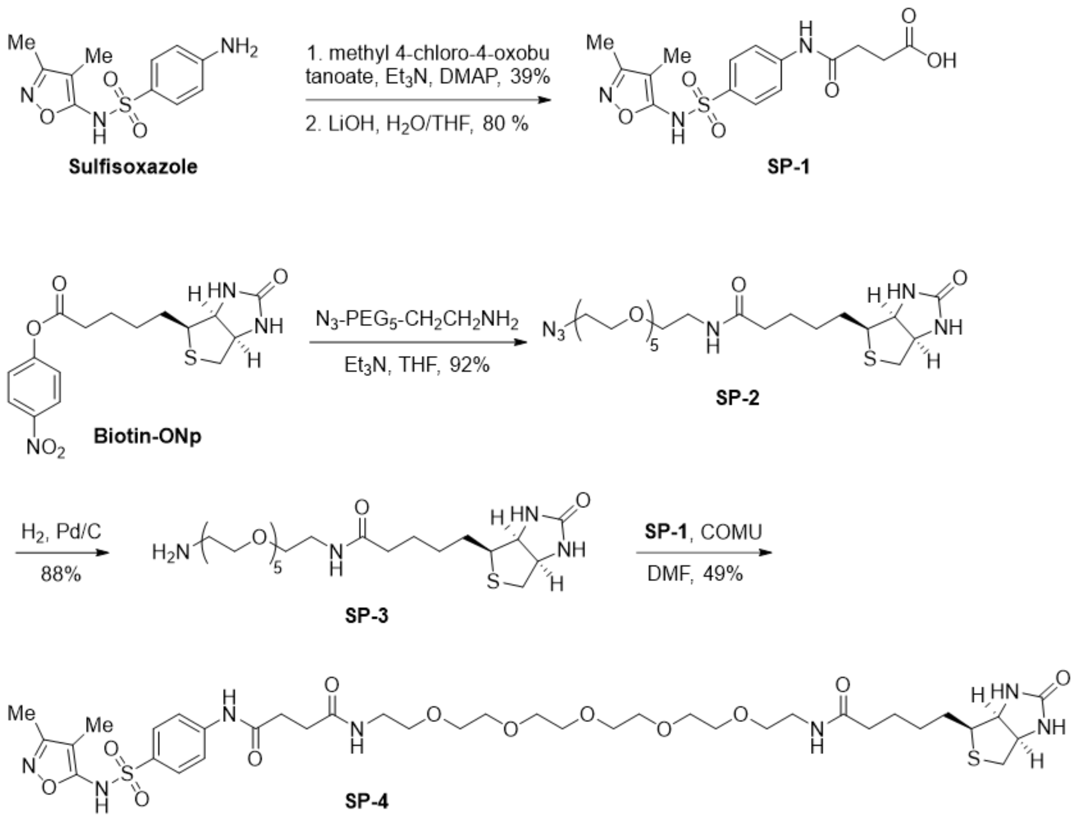

Supplementary Figure 14. Full images of immunoblots.

Full uncropped images and signals detected by immunoblotting, with the regions used in the corresponding main display items indicated by red frames.

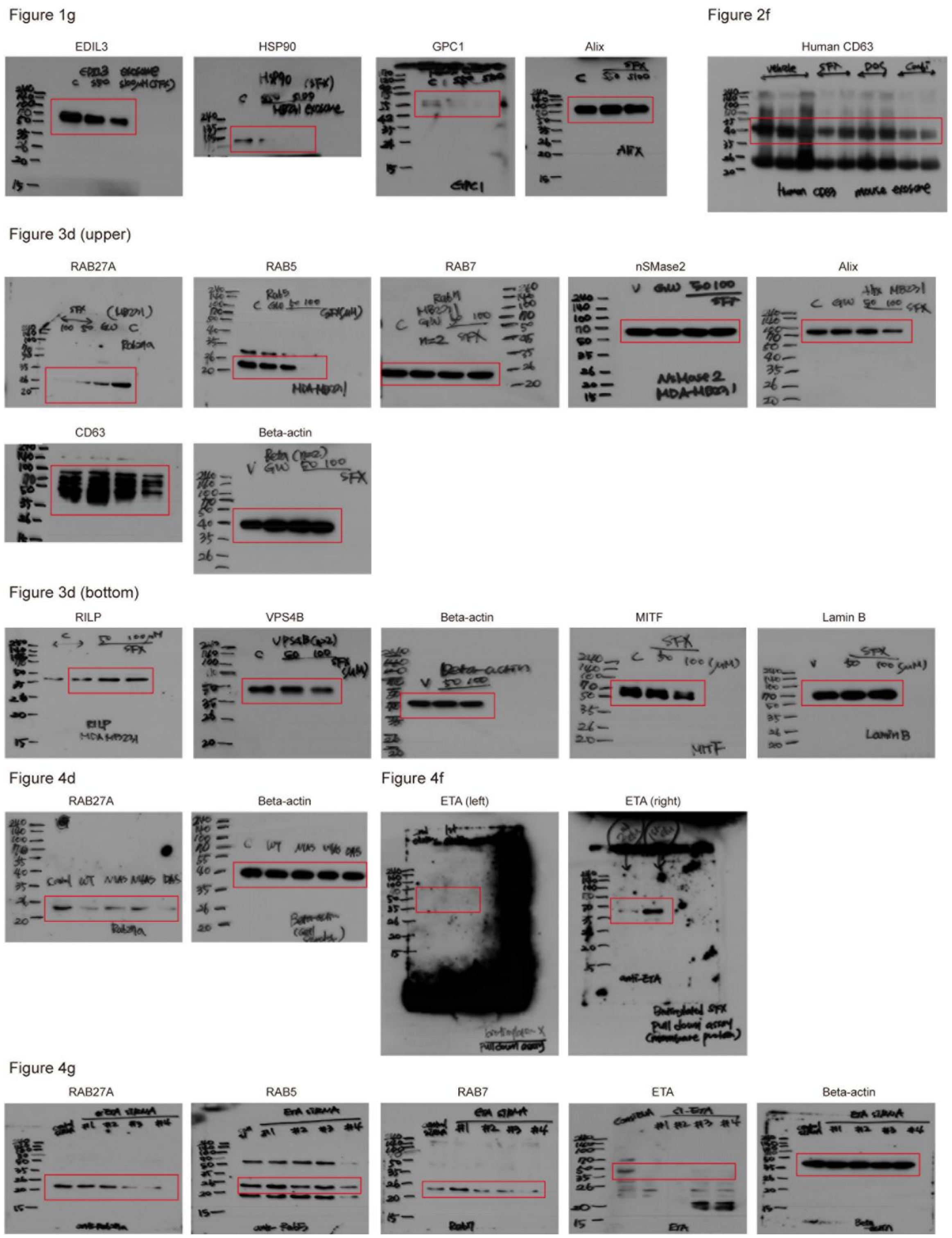

Figure 4h

si-AGTR1 transfection | si-CA13 transfection

RAB27A | AGTR1 | Beta-actin | RAB27A | CA13 | Beta-actin

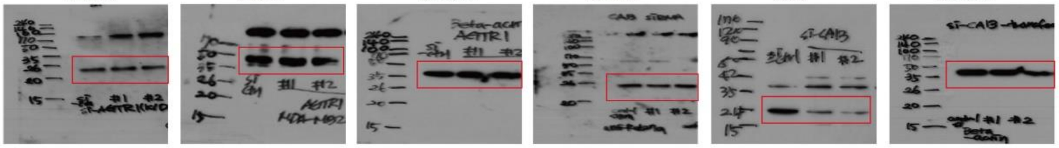

si-KMO transfection

RAB27A | KMO | Beta-actin

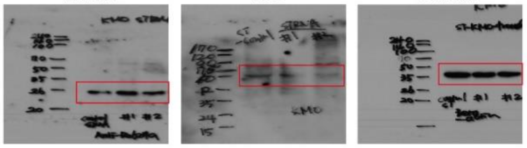

Figure 4j

ETA | ET1 | ET2 | Beta-actin

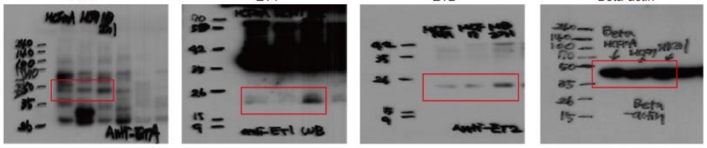

Figure 5b

ETA | RAB27A | RAB5 | RAB7 | Beta-actin

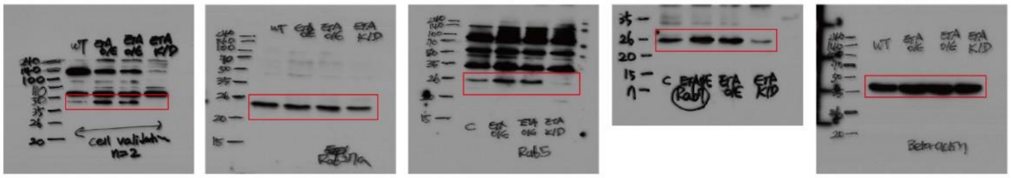

Figure 5b (cont.)

CD63 | Beta-actin

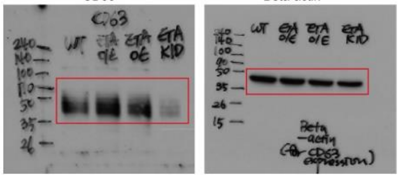

Figure 5g

Human-CD63

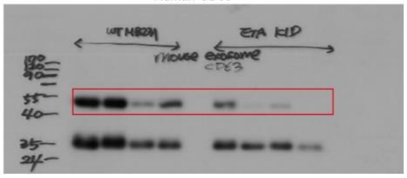

Figure 6b

LAMP1 | Beta-actin

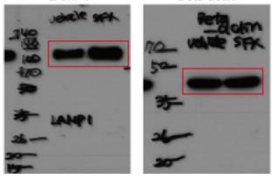

Figure 7c

LAMP1 | Beta-actin | LAMP1 | Beta-actin

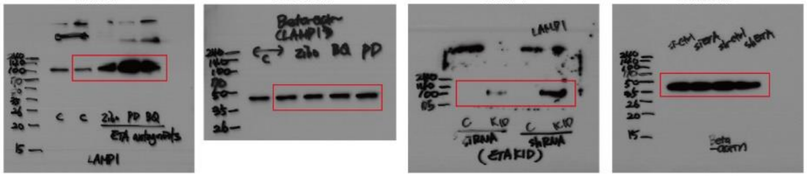

Supplementary figure 1c

copGFP | CD63

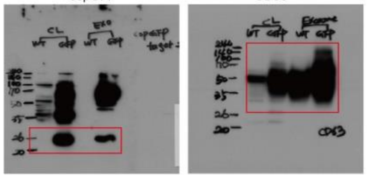

Supplementary figure 3b

CD9 | CD81 | CD63 | CD9 | CD81

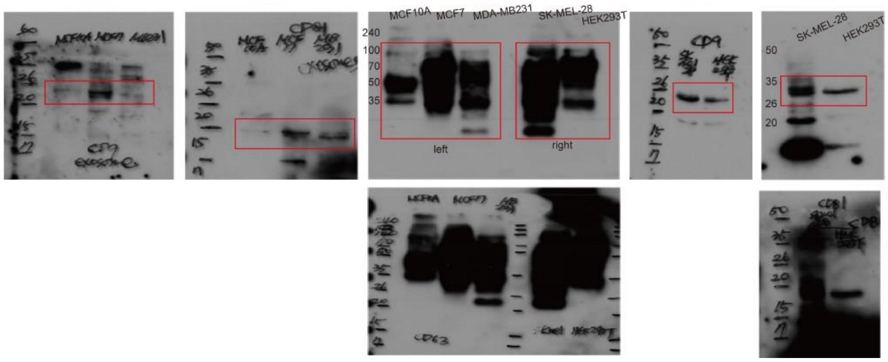

Supplementary figure 3f (left)

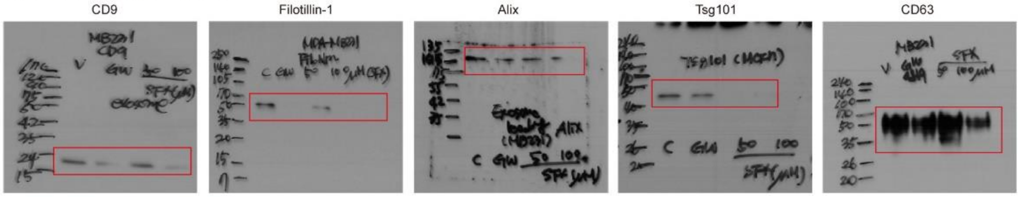

Supplementary figure 3f (right)

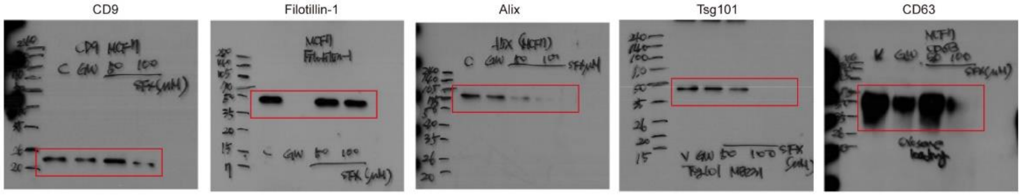

Supplementary figure 3g

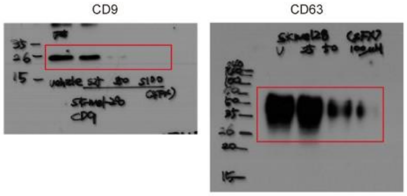

Supplementary figure 3h

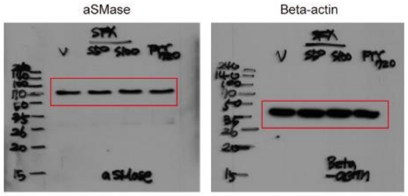

Supplementary figure 7b

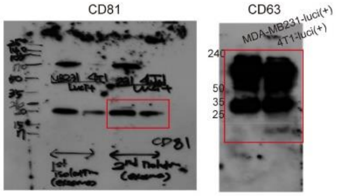

Supplementary figure 9a

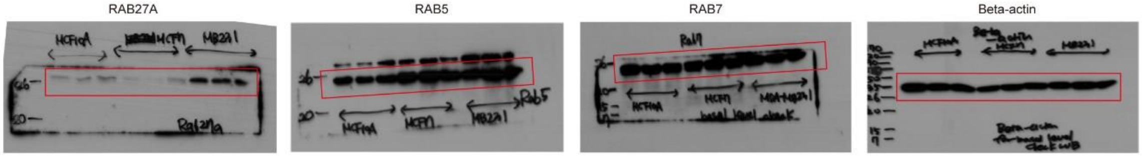

Supplementary figure 9b

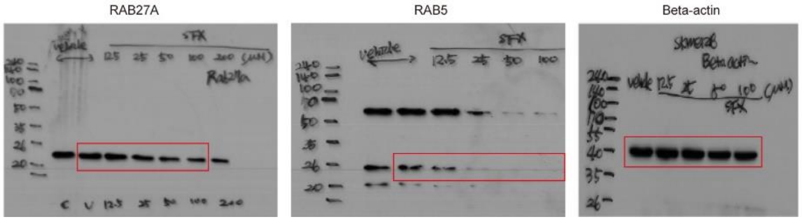

Supplementary figure 9c

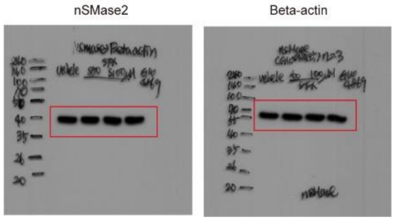

Supplementary Table 1. List of primer sequences used for reverse-transcription-polymerase chain reaction (RT-PCR)

Primer sequences used for reverse-transcription-polymerase chain reaction (RT-PCR) (5’ to 3’)

Gene	Forward primer	Reverse primer
RAB27A	AGT TGA TGG AGC GAA CTG CT	CCC TAC ACC AGA GTC TCC CA
RAB5A	ACG GGC CAA ATA CGG GAA AT	TCA AAC TTT ACC CCA ATG GTA CT
RAB7	GAT GGT GGA TGA CAG GCT AGT	CGA GAG ACT GGA ACC GTT CCT
RILP	CAC CCC CGG AGT CCA AAA TA	CAC AGC CAG ACC CCT AAG TC
MITF	AAG GGC TTG CAG AAC ACC TTA	GCT GGT TTG GAC ATG GCA AG
VPS4B	GAA ATC GGA AGT GCC CGG AG	CCA TCA CTG TCA TTC CCC TTC TC
Beta-actin	TTC CTG GGC ATG GAG TCC TGT GG	CGC CTA GAA GCA TTT GCG GTG G

Supplementary Table 2. List of miRNA sequences used for miRNA expression analysis

miRNA sequence used for miRNA expression analysis

miRNA	miRBase Accession Number	Mature miRNA sequence	Assay ID
**miR-21**	MIMAT0000076	UAGCUUAUCAGACUGAUGUUGA	000397
**miR-23b**	MIMAT0000530	UAGCUUAUCAGACUGAUGUUGA	002126
**miR-320b**	MIMAT0005792	AAAAGCUGGGUUGAGAGGGCAA	002844
**miR-182**	MIMAT0000259	UUUGGCAAUGGUAGAACUCACACU	002334
**miR-10b**	MIMAT0004556	ACAGAUUCGAUUCUAGGGGAAU	002315
**miR-105**	MIMAT0004516	ACGGAUGUUUGAGCAUGUGCUA	002168
**miR-122**	MIMAT0000421	UGGAGUGUGACAAUGGUGUUUG	002245
**miR-503**	MIMAT0022925	GGGGUAUUGUUUCCGCUGCCAGG	001048
**U6 snRNA**		GTGCTCGCTTCGGCAGCACATATACTAAAATTGGAAVGATAVAGAGAAGATTAGCATGGCCCCTGCGCAAGGATGACACGCAAATTCGTGAAGCGTTCCATATTTT	001973

Supplementary Table 3. List of siRNA sequences used for RNAi interference analysis

The siRNA sequence used for RNAi interference analysis

Target		Target sequence	Cat.#
ETA	#1	GUGAUAAUCCUGAGAGAUA	J-005485-05
	#2	GACAAGAACCGAUGUGAAU	J-005485-06
	#3	GCACUGGUUGGAUGUGUAA	J-005485-07
	#4	CCUCUGCGCUCUUAGUGUU	J-005485-08
AGTR1	#1	UGGAAGGCAUAAUUACAUA	J-005428-07
	#2	CCUGUACGCUAGUGUGUUU	J-005428-08
KMO	#1	UGGAUUAACAUUCGAGGAA	J-009897-09
	#2	CGUUAUAUAGCAUCGACAA	J-009897-10
CA13	#1	GGUAAACAAACUCGAUUCA	J-027325-09
	#2	CUGCCGUGUUGAUAGAAUA	J-027325-10
