# Supplementary material for: Sulfisoxazole inhibits the secretion of small extracellular vesicles by targeting the endothelin receptor A
Source: Nat Commun. 2019 Mar 27;10:1387. doi: 10.1038/s41467-019-09387-4 (PMC6437193; doi:10.1038/s41467-019-09387-4)
Supplement: Supplementary file 2 — Reporting Summary [file 41467_2019_9387_MOESM2_ESM.pdf]

nature research

Corresponding author(s): NCOMMS-18-25923A

Last updated by author(s): Feb 19, 2019

Reporting Summary

Nature Research wishes to improve the reproducibility of the work that we publish. This form provides structure for consistency and transparency in reporting. For further information on Nature Research policies, see Authors & Referees and the Editorial Policy Checklist.

Statistics

For all statistical analyses, confirm that the following items are present in the figure legend, table legend, main text, or Methods section.

n/a	Confirmed	
☐	☒	The exact sample size (*n*) for each experimental group/condition, given as a discrete number and unit of measurement
☐	☒	A statement on whether measurements were taken from distinct samples or whether the same sample was measured repeatedly
☐	☒	The statistical test(s) used AND whether they are one- or two-sided<br>*Only common tests should be described solely by name; describe more complex techniques in the Methods section.*
☐	☒	A description of all covariates tested
☐	☒	A description of any assumptions or corrections, such as tests of normality and adjustment for multiple comparisons
☐	☒	A full description of the statistical parameters including central tendency (e.g. means) or other basic estimates (e.g. regression coefficient) AND variation (e.g. standard deviation) or associated estimates of uncertainty (e.g. confidence intervals)
☐	☒	For null hypothesis testing, the test statistic (e.g. *F*, *t*, *r*) with confidence intervals, effect sizes, degrees of freedom and *P* value noted<br>*Give P values as exact values whenever suitable.*
☒	☐	For Bayesian analysis, information on the choice of priors and Markov chain Monte Carlo settings
☒	☐	For hierarchical and complex designs, identification of the appropriate level for tests and full reporting of outcomes
☒	☐	Estimates of effect sizes (e.g. Cohen's *d*, Pearson's *r*), indicating how they were calculated

Our web collection on statistics for biologists contains articles on many of the points above.

Software and code

Policy information about availability of computer code

Data collection	(1) NTA software (version 2.0, nanosight); (2) Affymetrix GeneChip Command Console software; (3) Affymetrix Power Tolls (APT) software; (4) PEAKs studio version 10.0; (5) MathIQTM
Data analysis	(1) ImageJ software; (2) Zeiss LSM Image Browser; (3) GraphPad Prism 6

For manuscripts utilizing custom algorithms or software that are central to the research but not yet described in published literature, software must be made available to editors/reviewers. We strongly encourage code deposition in a community repository (e.g. GitHub). See the Nature Research guidelines for submitting code & software for further information.

Data

Policy information about availability of data

All manuscripts must include a data availability statement. This statement should provide the following information, where applicable:

- Accession codes, unique identifiers, or web links for publicly available datasets
- A list of figures that have associated raw data
- A description of any restrictions on data availability

All microarray data that support the findings of this research have been deposited in the Gene Expression Omnibus (GEO) and are accessible through the GEO accession number GSE117991 (mRNA microarray) and GSE124320 (miRNA microarray). The mass spectrometry proteomics data has been deposited to the ProteomeXchange Consortium via the PRIDE with the dataset identifier PXD012689. All other relevant data of this study are available from the corresponding authors upon reasonable request.

Field-specific reporting

Please select the one below that is the best fit for your research. If you are not sure, read the appropriate sections before making your selection.

☒ Life sciences ☐ Behavioural & social sciences ☐ Ecological, evolutionary & environmental sciences

For a reference copy of the document with all sections, see nature.com/documents/nr-reporting-summary-flat.pdf

Life sciences study design

All studies must disclose on these points even when the disclosure is negative.

Sample size	Sample size for each experiment is indicated in the figures or corresponding figure legends. The sample size of animal study was chosen based on previous experience in the lab.
Data exclusions	No data were excluded from the analysis.
Replication	All replicates reported in the manuscript are biological replicates. All the statistics reported in the manuscript are based on at least 3 independent replicates.
Randomization	The age matched animals were randomized into control and drug-treated groups
Blinding	The researchers were blinded to allocation during analysis and outcome assessment. Animal experiments were performed in a blinded fashion when possible.

Reporting for specific materials, systems and methods

We require information from authors about some types of materials, experimental systems and methods used in many studies. Here, indicate whether each material, system or method listed is relevant to your study. If you are not sure if a list item applies to your research, read the appropriate section before selecting a response.

Materials & experimental systems

n/a	Involved in the study	
☐	☒	Antibodies
☐	☒	Eukaryotic cell lines
☒	☐	Palaeontology
☐	☒	Animals and other organisms
☒	☐	Human research participants
☒	☐	Clinical data

Methods

n/a	Involved in the study	
☒	☐	ChIP-seq
☒	☐	Flow cytometry
☒	☐	MRI-based neuroimaging

Antibodies

Antibodies used

The following primary antibodies were used for western blotting. We summarized antigen, dilution factor, host, supplier, catalog number and clone information.

(1) anti-CD63: 1:1000, rabbit, abcam, ab68418;
(2) anti-CD9: 1:1000, mouse, abcam, ab2215, clone: MEM61;
(3) anti-CD81: 1:1000, rabbit, abcam, ab109201, clone: EPR4244;
(4) anti-Alix: 1:1000, mouse, abcam, ab56932, clone: 3C4;
(5) anti-Tsg101: 1:1000, rabbit, abcam, ab30871;
(6) anti-Filotillin-1: 1:1000, rabbit, Cell Signaling Technology, #3253;
(7) anti-LAMP1: 1:1000, mouse, abcam, ab25630, clone: H4A3;
(8) anti-RILP: 1:1000, rabbit, abcam, ab128616;
(9) anti-beta-actin: 1:3000, rabbit, Cell Signaling Technology, #12620, clone: D6A8;
(10) anti-RAB27A: 1:1000, mouse, abcam, ab55667, clone: unknown clone;
(11) anti-RAB5: 1:1000, rabbit, abcam, ab18211;
(12) anti-RAB7: 1:1000, mouse, abcam, ab50533, clone: Rab7-117;
(13) anti-EDIL3: 1:1000, mouse, abcam, ab88667, clone: unknown clone;
(14) anti-Glypican-1: 1:1000, mouse, Thermo Fisher, PA5-28055;
(15) anti-HSP90a: 1:1000, rabbit, Cell Signaling Technology, #8165, clone: D1A7;
(16) anti-ASMase: 1:1000, rabbit, Cell Signaling Technology, #3687;
(17) anti-nSMase2: 1:1000, mouse, abcam, ab68735;
(18) anti-Endothelin receptor type A: 1:2500, rabbit, abcam, ab117521;
(19) anti-Angiotensin II type I receptor: 1:1000, rabbit, abcam, ab18801;
(20) anti-Carbonic anhydrases-13: 1:1000, rabbit, abcam, ab135986;

(21) anti-kynurenine 3-monooxygenase: 1:1000, rabbit, abcam, ab130959;
(22) anti-Endothelin-1: 1:1000, mouse, abcam, ab2786, clone: TR.ET.48.5;
(23) anti-Endothelin-2: 1:1000, mouse, Santacruz biotechnology, sc-293248, clone: 3B4-1C5;
(24) anti-VPS4B: 1:1000, rabbit, abcam, ab102687;
(25) anti-MITF: 1:1000, rabbit, Cell Signaling Technology, #12590, clone: D5G7V;
(26) anti-Lamin B: 1:1000, rabbit, Cell Signaling Technology, #13435, clone: D9V6H;
(27) Anti-human CD63: 1:1000, mouse, Cosmo Bio Co.,Ltd., SHI-EXO-M02, clone: 8A12.

Validation

All antibodies were obtained from commercial companies, and validated by the data sheets of the manufacturer or references listed below.
(1) anti-CD63 (1:1000, ab68418, abcam) Sung BH et al. Directional cell movement through tissues is controlled by exosome secretion. Nat Commun 6:7164 (2015);
(2) anti-CD9 (1:1000, ab2215, abcam, Clone: MEM61) Vallabhaneni KC et al. Extracellular vesicles from bone marrow mesenchymal stem/stromal cells transport tumor regulatory microRNA, proteins, and metabolites. Oncotarget 6:4953-67 (2015);
(3) anti-CD81 (1:1000, ab109201, abcam, Clone: EPR4244) Dong H et al. Exosome-mediated transfer of lncRNA-SNHG14 promotes trastuzumab chemoresistance in breast cancer. Int J Oncol 53:1013-1026 (2018);
(4) anti-Alix (1:1000, ab56932, abcam, Clone: 3C4) Lee CH et al. Discovery of a diagnostic biomarker for colon cancer through proteomic profiling of small extracellular vesicles. BMC cancer 18:1058 (2018);
(5) anti-Tsg101 (1:1000, ab30871, abcam) Kooijmans SA et al. Display of GPI-anchored anti-EGFR nanobodies on extracellular vesicles promotes tumour cell targeting. J Extracell Vesicles 5:31053 (2016);
(6) anti-Filotillin-1 (1:1000, #3253, Cell Signaling Technology) Boulter E et al. Cell metabolism regulates integrin mechanosensing via an SLC3A2-dependent sphingolipid biosynthesis pathway. Nat. Commun 9:4862 (2018);
(7) anti-LAMP1 (1:1000, ab128616, abcam) Poillet-Perez L et al. GABARAPL1 tumor suppressive function is independent of its conjugation to autophagosomes in MCF-7 breast cancer cells. Oncotarget 8:55998-56020 (2017);
(8) anti-RILP (1:1000, ab128616, abcam) Modica et al. Rab7 palmitoylation is required for efficient endosome-to-TGN trafficking. J Cell Sci 130:2579-2590;
(9) anti-beta-actin (1:3000, #12620, Cell Signaling Technology, Clone: D6A8) Duan R et al. Effects of Vaspin on Insulin Resistance in Rats and Underlying Mechanisms. Sci Rep 10:13542 (2018);
(10) anti-RAB27A (1:1000, ab55667, abcam, Clone: unknown clone) Ostenfeld MS et al. Cellular disposal of miR23b by RAB27-dependent exosome release is linked to acquisition of metastatic properties. Cancer Res 74:5758-71 (2014);
(11) anti-RAB5 (1:1000, ab18211, abcam) Lim D et al. The hepcidin-ferroportin axis controls the iron content of Salmonella-containing vacuoles in macrophages. Nat commun 9:2091 (2018);
(12) anti-RAB7 (1:1000, ab50533, abcam, Clone: Rab7-117) Yamano K et al. Endosomal Rab cycles regulate Parkin-mediated mitophagy. Elife 7:N/A (2018);
(13) anti-EDIL3 (1:1000, ab88667, abcam, Clone: unknown clone) Moon PG et al. Identification of Developmental Endothelial Locus-1 on Circulating Extracellular Vesicles as a Novel Biomarker for Early Breast Cancer Detection. Clin Cancer Res 22:1757-66 (2016);
(14) anti-Glypican-1 (1:1000, PA5-28055 Thermo Fisher) Melo SA et al. Glypican-1 identifies cancer exosomes and detects early pancreatic cancer. Nature 523:177-82 (2015);
(15) anti-HSP90a (1:1000, #8165, Cell Signaling Technology, Clone: D1A7) Huang Z et al. Hyperthermia enhances 17-DMAC efficacy in hepatocellular carcinoma cells with aggravated DNA damage and impaired G2/M transition. Sci Rep 6:38072 (2016);
(16) anti-ASMase (1:1000, #3687, Cell Signaling Technology) Waster P et al. Extracellular vesicles are transferred from melanocytes to keratinocytes after UVA irradiation. Sci. Rep 6:27890 (2016);
(17) anti-nSMase2 (1:1000, ab68735, abcam) https://www.abcam.com/nsmase2-antibody-ab68735.html;
(18) anti-Endothelin receptor type A (1:2500, ab117521, abcam) Choy, MK et al. Promoter interactome of human embryonic stem cell-derived cardiomyocytes connects GWAS regions to cardiac gene networs. Nat commun 9:2526 (2018);
(19) anti-Angiotensin II type I receptor (1:1000, ab18801, abcam) Cops J et al. Selective abdominal venous congestion induces adverse renal and hepatic morphological and functional alterations despite a preserved cardiac function. Sci Rep 8:17757 (2018);
(20) anti-Carbonic anhydrases-13 (1:1000, ab135986, abcam) Hulikova A et al. Intracellular carbonic anhydrases activity sensitizes cancer cell pH signaling to Dynamic changes in CO2 partial pressure. J Biol Chem 289:25418-25430 (2014);
(21) anti-kynurenine 3-monooxygenase (1:1000, ab130959, abcam) https://www.abcam.com/kynurenine-3-monooxygenase-antibody-ab130959.html;
(22) anti-Endothelin-1 (1:1000, ab2786, abcam, Clone: TR.ET.48.5) Mahmoud AM et al. Hyperinsulinemia augments endothelin-1 protein expression and impairs vasodilation of human skeletal muscel arterioles. Physiol Rep 4:e12895 (2016);
(23) anti-Endothelin-2 (1:1000, sc-293248, Santacruz biotechnology, Clone: 3B4-1C5) https://datasheets.scbt.com/sc-293248.pdf;
(24) anti-VPS4B (1:1000, ab102687, abcam) Shtanko, O et al. Crimean-Congo hemorrhagic fever virus entry into host cells occurs through the multivesicular body and requires ESCRT regulators. PLoS Pathog 10:e1004390 (2014);
(25) anti-MITF (1:1000, #12590, Cell Signaling Technology, Clone: D5G7V) Natale CA et al. Sex steroids regulate skin pigmentation through nonclassical membrane-bound receptors. Elife 5:e15104 (2016);
(26) anti-Lamin B (1:1000, #13435, Cell Signaling Technology, Clone: D9V6H) Ni Y et al. Dephosphorylated Polymerase I and Transcript Release Factor Prevents Allergic Asthma Exacerbations by Limiting IL-33 Release. Front Immunol 9:1422 (2018);

For the detection of specific human-cell derived sEV, the following primary antibody was used.
(27) Anti-human CD63 (1:1000, SHI-EXO-M02, Cosmo Bio Co.,Ltd. Clone: 8A12) Nishida-Aoki N et al. Disruption of Circulating Extracellular Vesicles as a Novel Therapeutic Strategy against Cancer Metastasis. Mol Ther 25:181-191 (2017).

Eukaryotic cell lines

Policy information about cell lines

Cell line source(s)

MCF10A, MDA-MB231, SK-MEL-28, HEK293T and MCF7 cells were obtain from ATCC (American Type Culture Collection)

Authentication	All cells were originally obtained from the ATCC cell repository
Mycoplasma contamination	All cell lines were tested for mycoplasma contamination by PCR genotyping
Commonly misidentified lines (See ICLAC register)	No commonly misidentified cell lines were used.

Animals and other organisms

Policy information about studies involving animals; ARRIVE guidelines recommended for reporting animal research

Laboratory animals	Mouse (Mus musculus);<br>BALB/cAnN/CrljOri-nu/nu (Balb/c nude), 5 weeks; Balb/cAnN/CrljOri (Balb/c WT), 5 weeks; ICR mice 7 weeks
Wild animals	This study did not involve wild animals
Field-collected samples	The acquisition, care, housing, use, and disposition of animals in research were in compliance with applicable federal, state, and local, laws and regulations.
Ethics oversight	All animal research was performed in accordance with protocols approved by the Kyungpook National University (KNU) Institutional Animal Care and Use Committes (IACUCs. Approved number: 2017-0146) and approved by Chung-Ang University (CAU).

Note that full information on the approval of the study protocol must also be provided in the manuscript.
